# Safety, pharmacokinetics, and antimalarial activity of the novel plasmodium eukaryotic translation elongation factor 2 inhibitor M5717: a first-in-human, randomised, placebo-controlled, double-blind, single ascending dose study and volunteer infection study

**DOI:** 10.1016/S1473-3099(21)00252-8

**Published:** 2021-12

**Authors:** James S McCarthy, Özkan Yalkinoglu, Anand Odedra, Rebecca Webster, Claude Oeuvray, Aliona Tappert, Deon Bezuidenhout, Marla J Giddins, Satish K Dhingra, David A Fidock, Louise Marquart, Lachlan Webb, Xiaoyan Yin, Akash Khandelwal, Wilhelmina M Bagchus

**Affiliations:** a QIMR Berghofer Medical Research Institute, Brisbane, QLD, Australia; b the healthcare business of Merck KGaA, Darmstadt, Germany; c The Global Health Institute of Merck (an affiliate of Merck KGaA), Eysin, Switzerland; d Merck (an affiliate of Merck KGaA), Modderfontein, South Africa; e Department of Microbiology and Immunology, Columbia University Irving Medical Center, New York, NY, USA; f Division of Infectious Diseases, Department of Medicine, Columbia University Irving Medical Center, New York, NY, USA; g Global Statistics for NDD, Immunology, Endocrinology, Fertility & Others, EMD Serono, Billerica, MA, USA; h Merck Institute for Pharmacometrics, Lausanne, Switzerland; i The Peter Doherty Institute for Infection and Immunity, The University of Melbourne and the Royal Melbourne Hospital, Melbourne, VIC, Australia; j Liverpool School of Tropical Medicine, Liverpool, UK

## Abstract

**Background:**

M5717 is the first plasmodium translation elongation factor 2 inhibitor to reach clinical development as an antimalarial. We aimed to characterise the safety, pharmacokinetics, and antimalarial activity of M5717 in healthy volunteers.

**Methods:**

This first-in-human study was a two-part, single-centre clinical trial done in Brisbane, QLD, Australia. Part one was a double-blind, randomised, placebo-controlled, single ascending dose study in which participants were enrolled into one of nine dose cohorts (50, 100, 200, 400, 600, 1000, 1250, 1800, or 2100 mg) and randomly assigned (3:1) to M5717 or placebo. A sentinel dosing strategy was used for each dose cohort whereby two participants (one assigned to M5717 and one assigned to placebo) were initially randomised and dosed. Randomisation schedules were generated electronically by independent, unblinded statisticians. Part two was an open-label, non-randomised volunteer infection study using the *Plasmodium falciparum* induced blood-stage malaria model in which participants were enrolled into three dose cohorts. Healthy men and women of non-childbearing potential aged 18–55 years were eligible for inclusion; individuals in the volunteer infection study were required to be malaria naive. Safety and tolerability (primary outcome of the single ascending dose study and secondary outcome of the volunteer infection study) were assessed by frequency and severity of adverse events. The pharmacokinetic profile of M5717 was also characterised (primary outcome of the volunteer infection study and secondary outcome of the single ascending dose study). Parasite clearance kinetics (primary outcome of the volunteer infection study) were assessed by the parasite reduction ratio and the corresponding parasite clearance half-life; the incidence of recrudescence up to day 28 was determined (secondary outcome of the volunteer infection study). Recrudescent parasites were tested for genetic mutations (exploratory outcome). The trial is registered with ClinicalTrials.gov (NCT03261401).

**Findings:**

Between Aug 28, 2017, and June 14, 2019, 221 individuals were assessed for eligibility, of whom 66 men were enrolled in the single ascending dose study (eight per cohort for 50–1800 mg cohorts, randomised three M5717 to one placebo, and two in the 2100 mg cohort, randomised one M5717 to one placebo) and 22 men were enrolled in the volunteer infection study (six in the 150 mg cohort and eight each in the 400 mg and 800 mg cohorts). No adverse event was serious; all M5717-related adverse events were mild or moderate in severity and transient, with increased frequency observed at doses above 1250 mg. In the single ascending dose study, treatment-related adverse events occurred in three of 17 individuals in the placebo group; no individual in the 50 mg, 100 mg, or 200 mg groups; one of six individuals in each of the 400 mg, 1000 mg, and 1250 mg groups; two of six individuals in the 600 mg group; and in all individuals in the 1800 mg and 2100 mg groups. In the volunteer infection study, M5717-related adverse events occurred in no participants in the 150 mg or 800 mg groups and in one of eight participants in the 400 mg group. Transient oral hypoesthesia (in three participants) and blurred vision (in four participants) were observed in the 1800 mg or 2100 mg groups and constituted an unknown risk; thus, further dosing was suspended after dosing of the two sentinel individuals in the 2100 mg cohort. Maximum blood concentrations occurred 1–7 h after dosing, and a long half-life was observed (146–193 h at doses ≥200 mg). Parasite clearance occurred in all participants and was biphasic, characterised by initial slow clearance lasting 35–55 h (half-life 231·1 h [95% CI 40·9 to not reached] for 150 mg, 60·4 h [38·6 to 138·6] for 400 mg, and 24·7 h [20·4 to 31·3] for 800 mg), followed by rapid clearance (half-life 3·5 h [3·1 to 4·0] for 150 mg, 3·9 h [3·3 to 4·8] for 400 mg, and 5·5 h [4·8 to 6·4] for 800 mg). Recrudescence occurred in three (50%) of six individuals dosed with 150 mg and two (25%) of eight individuals dosed with 400 mg. Genetic mutations associated with resistance were detected in four cases of parasite recrudescence (two individuals dosed with 150 mg and two dosed with 400 mg).

**Interpretation:**

The safety, pharmacokinetics, and antimalarial activity of M5717 support its development as a component of a single-dose antimalarial combination therapy or for malaria prophylaxis.

**Funding:**

Wellcome Trust and the healthcare business of Merck KGaA, Darmstadt, Germany.

## Introduction

The decline in the worldwide burden of malaria achieved earlier in the 21st century has now plateaued,^1^ a situation exacerbated by the emergence of resistance to first-line artemisinin-based combination therapies in southeast Asia.^2^ An estimated 229 million cases and 409 000 deaths were attributable to the disease in 2019.^1^ The development of new antimalarial treatments is one important component of the fight to reduce malaria morbidity and mortality. The ideal characteristics of new antimalarials include an ability to reduce the parasite burden rapidly; a long duration of activity to avoid the requirement for multiple dosing; activity against multiple parasite lifecycle stages, including dormant liver stages and transmissible gametocytes; an ability to be used for chemoprotection; and a novel mode of action to avoid cross-resistance with existing antimalarials.^3^ The combination of two or more compounds is probably necessary to achieve these properties and is considered an important strategy to minimise the risk of resistance emerging to a novel compound. Finally, as the affordability of any new antimalarial treatment is an important factor, the cost of manufacturing a candidate compound must be considered before investing in clinical development.^3^

M5717 (formerly DDD107498) is a novel antimalarial compound discovered through a phenotypic screen, and it was shown to exhibit nanomolar potency against *Plasmodium falciparum*.^4^, ^5^ The molecular target of M5717 was identified as translation elongation factor 2 (eEF2), which is essential for protein synthesis due to its role in GTP-dependent translocation of the ribosome along mRNA.^6^ Preclinical in-vitro and in-vivo analyses indicated that M5717 has high selectivity for plasmodium eEF2 and activity against multiple *Plasmodium* spp lifecycle stages (including liver-stage and both asexual and sexual blood-stage parasites). These studies also indicated an acceptable safety profile and good pharmacokinetic properties.^4^ Collectively, these properties indicated the potential for the compound to offer single-dose cure, reduce transmission, and achieve chemoprotection. Furthermore, initial cost-of-goods estimates and likely human-dose projections suggested an acceptable cost per treatment.^4^ Together, the results of preclinical work indicated that M5717 is a promising candidate to advance to clinical development as a new antimalarial treatment.

This report describes the first in-human clinical trial to characterise the safety, tolerability, pharmacokinetics, and antimalarial activity of M5717 in healthy volunteers. Our study was undertaken in two parts. The first part was a randomised single ascending dose study with oral administration of M5717 or matching placebo. The second part was a volunteer infection study using the induced blood-stage malaria model,^7^ in which malaria-naive volunteers were inoculated with *P falciparum*-infected erythrocytes and subsequently administered a single oral dose of M5717. This work represents the first clinical investigation of an antimalarial compound targeting plasmodium eEF2.

**Research in context**

**Evidence before this study**

New antimalarials with novel mechanisms of action are needed to combat the emergence of drug resistance and to progress towards malaria elimination. M5717 (formerly DDD107498) is a new antimalarial candidate that inhibits plasmodium translation elongation factor 2, an enzyme essential for protein synthesis. We searched PubMed between Jan 1, 2010, and Feb 19, 2021, using the terms “plasmodium translation elongation factor 2 inhibitor”, “M5717”, and “DDD107498”. Preclinical studies showed that M5717 has potent antimalarial activity towards multiple *Plasmodium* spp lifecycle stages in vitro and in vivo and good pharmacokinetic properties amenable to use as a drug and predicted an acceptable safety profile in humans. Together these findings supported the progression of M5717 into clinical development.

**Added value of this study**

This is the first in-human clinical trial of M5717. This study integrates a single ascending dose study with a volunteer infection study using the *Plasmodium falciparum* induced blood-stage malaria model to characterise the safety, pharmacokinetics, and antimalarial activity of M5717 in healthy adults. Our results showed that M5717 is well tolerated in humans up to and including a dose of 1250 mg, with the incidence of M5717-related adverse events remaining low up to this dose, whereas mild-to-moderate and transient neurological adverse events were observed with dosing at 1800 mg and 2100 mg. M5717 exhibits a pharmacokinetic profile characterised by a long half-life (146–193 h at doses ≥200 mg). Single oral doses of M5717 (150 mg, 400 mg, or 800 mg) resulted in initial clearance of asexual blood-stage parasitaemia with a biphasic profile, characterised by an initial period of slow clearance lasting 35–55 h, followed by a rapid clearance phase. Genetic mutations associated with resistance were detected in recrudescent parasites in four participants.

**Implications of all the available evidence**

M5717 is well tolerated in healthy adults at doses that clear blood-stage *P falciparum*, with the long half-life of the compound supporting the potential for single-dose administration with long-duration activity. Future studies to investigate combination therapies of M5717 with a partner drug exhibiting a distinct mode and rapid onset of action in treating clinical malaria are warranted, as are investigations into the use of M5717 for other indications, including chemoprotection and prevention of transmission.

## Methods

### Study design and participants

This study was planned to be done in three parts. Part A (done as planned) was a first-in-human, double-blind, randomised, placebo-controlled, single ascending dose study. Part B was planned to be a multiple ascending dose study that would be done if emerging data in part A indicated that it would not be possible to administer a tolerable single dose that would achieve exposure over 8 days at a concentration well above the minimum parasiticidal concentration estimated in preclinical studies.^4^ Data from the first four cohorts of the single ascending dose study resulted in a decision not to do the multiple ascending dose study (whose study design and methodology are therefore not presented here). Part C (done as planned) was an open-label, non-randomised volunteer infection study using the *P falciparum* induced blood-stage malaria model. The volunteer infection study began once the safety monitoring committee had reviewed data from the first four cohorts of the single ascending dose study and had determined that the exposure threshold had been reached.

Healthy men and women of non-childbearing potential aged 18–55 years were eligible for inclusion in both study parts (A and C); participants in the volunteer infection study were required to be malaria-naive (see appendix p 12 for full eligibility criteria). The study was done at Q-Pharm (Brisbane, QLD, Australia) following approval by the QIMR Berghofer Medical Research Institute Human Research Ethics Committee. All participants gave written informed consent before enrolment. The study is registered with ClinicalTrials.gov (NCT03261401).

### Randomisation and masking

Participants in the single ascending dose study were randomised within each dose cohort (50, 100, 200, 400, 600, 1000, 1250, 1800, or 2100 mg) to either M5717 or placebo in a 3:1 ratio. The randomisation schedules were generated electronically by independent, unmasked statisticians using SAS version 9.4. Participants and investigators were masked to the identity of the treatment from the time of randomisation until database lock. Treatment identity was concealed by the identical packaging, appearance, odour, and taste of M5717 and placebo.

### Procedures

After a screening period of 26 days, participants in the single ascending dose study were admitted to the clinic 2 days before dosing for baseline electrocardiograms (ECGs) on day −1. On dosing day (day 1), after randomisation, participants received a single oral dose of M5717 or placebo capsules (the healthcare business of Merck KGaA, Darmstadt, Germany) with 250 mL of water after fasting for at least 8 h; no food was allowed until 4 h after dosing. A sentinel dosing strategy was used for each dose cohort whereby two participants (one assigned to M5717 and one assigned to placebo) were initially randomised and dosed. The investigator reviewed blinded safety data up to at least 36 h after dosing before deciding whether to proceed with randomisation and dosing of the remaining six participants in the cohort. Participants were confined to the clinic until day 7 and returned as outpatients for follow-up visits until the end of the study.

The starting dose of M5717 used in the single ascending dose study (50 mg) was half of the maximum safe starting dose calculated in accordance with guidance from the US Food & Drug Administration.^8^ Dose selection for each subsequent cohort was decided by the safety monitoring committee based on safety data up to day 22 and pharmacokinetic data up to day 7. Since data from the first three cohorts (50, 100, and 200 mg) indicated that the observed terminal half-life of M5717 (133–145 h) was shorter than that predicted before the study (250 h), the safety monitoring committee approved a reduction of the duration of follow-up for participants in the remaining six cohorts from day 55 to day 44 for the end-of-study visit.

The volunteer infection study was done in three dose cohorts (150, 400, and 800 mg). After a screening period of 26 days, participants were inoculated intravenously with *P falciparum*-infected erythrocytes (containing approximately 2800 viable parasites) on day −8. Parasitaemia was monitored twice daily from the 4th day after the inoculation until the day of dosing with M5717 (day 1) on an outpatient basis by collecting blood samples and performing qPCR targeting the gene encoding *P falciparum* 18S rRNA.^9^ Participants were admitted to the clinic for M5717 dosing when parasitaemia was expected to be at least 5000 parasites per mL. A single oral dose of M5717 was administered as described for the single ascending dose study. Participants were confined to the clinic until day 4 and returned as outpatients for follow-up visits until the end of study visit on day 44. Participants received a standard curative course of artemether–lumefantrine treatment upon recrudescence or on day 21 if recrudescence had not occurred. Parasite DNA extracted from recrudescent *P falciparum* populations was subject to DNA sequence analysis of the *P falciparum eEF2* gene that encodes the drug target and resistance mediator^4^ to investigate potential mutations that could confer resistance to M5717 (appendix p 9).

The first cohort in the volunteer infection study was administered 400 mg M5717. This dose was selected on the basis of data obtained from a preclinical mouse efficacy model,^4^ as well as human pharmacokinetic data obtained from the first four cohorts of the single ascending dose study. A single 400 mg dose was estimated to maintain blood concentrations above the predicted minimum parasiticidal concentration for 8 days in the majority of participants. After review of the data obtained in the first cohort, the safety monitoring committee decided to lower the dose to 150 mg for the second cohort to better characterise the exposure–response relationship. After review of the data obtained in the 150 mg cohort, as well as pharmacokinetic and safety data from the 1000 mg cohort in the single ascending dose study, the safety monitoring committee decided that a third volunteer infection study cohort would proceed at a dose of 800 mg to further define the exposure–response relationship.

M5717 concentration in whole blood was determined using liquid chromatography tandem mass spectrometry (appendix p 11). A dried blood spot assay was done in the second and third cohorts of the volunteer infection study to assess the correlation of M5717 concentrations in whole blood with that in dried blood spots. Results of this analysis will be presented elsewhere. Blood sampling timepoints for M5717 concentration and parasitaemia measurements are specified in the appendix (p 15).

### Outcomes

The primary outcomes of the single ascending dose study and secondary outcomes of the volunteer infection study were the nature, incidence, and severity of adverse events, as well as the incidence of clinically significant changes and abnormalities in safety laboratory parameters (haematology, coagulation, biochemistry, and urinalysis), vital signs (body temperature, blood pressure, heart rate, and respiratory rate), and 12-lead ECGs. The medical assessment of adverse event severity was done in accordance with the Qualitative Toxicity Scale (mild=grade 1, moderate=grade 2, severe=grade 3, potentially life threatening=grade 4).^10^

The primary outcomes of the volunteer infection study and secondary outcomes of the single ascending dose study were the following pharmacokinetic parameters: maximum observed concentration (C_max_); time to reach C_max_ (t_max_); area under the concentration–time curve (AUC) from time 0 h (dosing) to the last sampling time at which the concentration was greater than or equal to the lower limit of quantification (LLOQ; AUC_0–t_); AUC from time 0 h extrapolated to infinity (AUC_0–∞_); AUC from time 0 h to 144 h after dosing (AUC_0–144h_); apparent terminal half-life (t_1/2_); apparent total clearance (CL/F); apparent volume of distribution (Vz/F); time at or above the minimum inhibitory concentration (MIC), estimated in mice to be 3 ng/mL (t_≥3 ng/mL_); and time at or above the minimum parasiticidal concentration, estimated in mice to be 10 ng/mL (t_≥10 ng/mL_).

Other primary outcomes of the volunteer infection study were the parasite reduction ratio (PRR) and the corresponding parasite clearance half-life (with the PRR expressed as the ratio of the parasite density decrease over a 48 h time period following M5717 dosing [PRR_48_]). Other secondary outcomes of the volunteer infection study were the malaria clinical score^11^ (to be presented elsewhere) and the incidence of recrudescence. Additional secondary outcomes for the single ascending dose study and the volunteer infection study were the MIC, minimum parasiticidal concentration, parasite clearance time, and lag phase (to be calculated using pharmacokinetic–pharmacodynamic modelling and presented elsewhere). Genetic mutations in recrudescent *P falciparum* populations conferring resistance to M5717 were an exploratory outcome.

### Statistical analysis

The intended sample size in the single ascending dose study (eight participants per cohort randomised 3:1 to M5717 or placebo) was based on general phase 1 trial experience and was considered appropriate to achieve the primary objective of this part of the study (determine the safety and tolerability of M5717). The intended sample size in the volunteer infection study (eight participants per cohort) was selected based on several previous induced blood-stage malaria studies that successfully characterised the parasite clearance kinetics of various antimalarial compounds.^11^, ^12^, ^13^, ^14^, ^15^

Non-compartmental pharmacokinetic analysis was done using Phoenix WinNonlin version 8.0. Pharmacodynamic analyses were done in R version 3.5.0. The PRR_48_ and parasite clearance half-life were estimated using the slope of the optimal fit for the log-linear relationship of the parasitaemia decay, as described previously,^16^ with modification due to the biphasic parasitaemia clearance profile observed. The pattern of parasite clearance was analysed at the participant level using segmented piecewise regression^17^ to estimate the clearance profile in two phases: the first phase corresponding to initial slow clearance, and the second to subsequent rapid clearance. Random-effects meta-analysis^18^ was used to calculate cohort-specific estimates of clearance (PRR_48_ and parasite clearance half-life) and the breakpoint (timepoint when the first clearance phase ends and the second clearance phase begins). Analyses were done in the per-protocol population.

### Role of the funding source

Authors employed by the healthcare business of Merck KGaA, Darmstadt, Germany, were involved in protocol development, study oversight, and data analysis and interpretation. The Wellcome Trust had no role in the study apart from representation on the safety monitoring committee.

## Results

**Figure 1:**
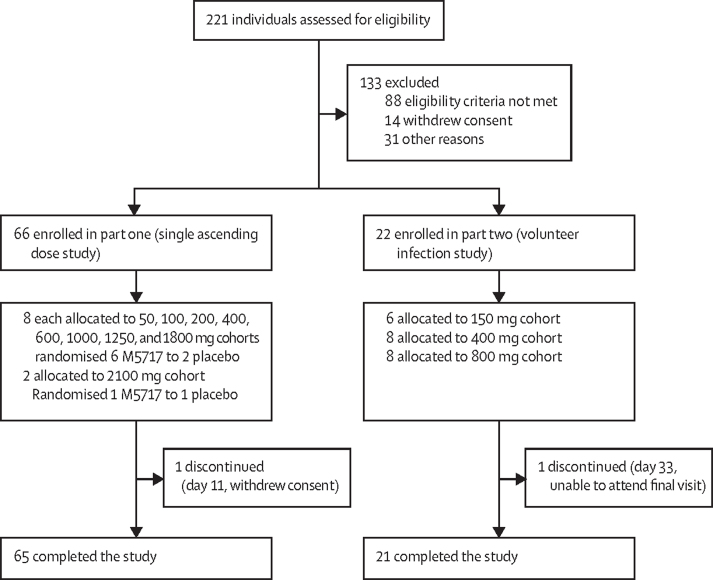
Trial profile In part one, single ascending doses of M5717 (50–2100 mg) were tested in nine cohorts. The volunteer infection study (part two) started after documentation of safety and pharmacokinetics data from the first four cohorts (up to the 400 mg dose cohort) in part one. The volunteer infection study consisted of three dose cohorts (150, 400, and 800 mg).

The study was done from Aug 28, 2017 (first participant consented), to June 14, 2019 (last participant's last visit). The dates for each cohort and database lock are presented in the appendix (p 16). In total, 221 volunteers were screened for eligibility, with 133 excluded (figure 1). 88 volunteers were enrolled, including 66 in the single ascending dose study and 22 in the volunteer infection study (figure 1). All participants enrolled in both parts were male and the majority self-selected their race as White (table 1). The first eight cohorts of the single ascending dose study (50–1800 mg) each contained eight participants, with six participants randomly assigned to M5717 and two randomly assigned to placebo. Several transient adverse events were observed following dosing of the two sentinel participants (one randomised to M5717 and one randomised to placebo) in the 2100 mg dose cohort, which resulted in the safety monitoring committee deciding not to enrol the remaining six participants in this cohort. Due to recruitment limitations, the 150 mg cohort of the volunteer infection study consisted of six participants instead of the planned eight. In total, two participants withdrew voluntarily before the end of study: one withdrew from the single ascending dose study (dosed with 600 mg M5717) on day 11 and one withdrew from the volunteer infection study (dosed with 800 mg M5717) on day 33. Available data from both participants were included in the analyses of study endpoints.

**Table 1:** Demographic profile of participants by dose cohort

		**Single ascending dose study**										**Volunteer infection study**		
		Placebo (N=17)	50 mg (N=6)	100 mg (N=6)	200 mg (N=6)	400 mg (N=6)	600 mg (N=6)	1000 mg (N=6)	1250 mg (N=6)	1800 mg (N=6)	2100 mg (N=1)	150 mg (N=6)	400 mg (N=8)	800 mg (N=8)
Age, years		28 (10·2)	28 (6·4)	31 (7·3)	30 (6·7)	27 (13·5)	25 (10·1)	28 (10·9)	26 (7·0)	30 (9·8)	43 (NC)	30 (11·2)	25 (5·9)	30 (9·4)
Race or ethnicity														
	White	13 (76%)	4 (67%)	4 (67%)	6 (100%)	5 (83%)	6 (100%)	2 (33%)	5 (83%)	4 (67%)	1 (100%)	4 (67%)	4 (50%)	8 (100%)
	Asian	2 (12%)	0	0	0	0	0	3 (50%)	0	1 (17%)	0	1 (17%)	1 (13%)	0
	Black or African American	1 (6%)	0	1 (17%)	0	0	0	0	0	1 (17%)	0	0	1 (13%)	0
	American Indian or Alaska Native	0	1 (17%)	1 (17%)	0	1 (17%)	0	0	0	0	0	1 (17%)	2 (25%)	0
	Native Hawaiian or Other Pacific Islander	0	1 (17%)	0	0	0	0	0	0	0	0	0	0	0
	Mixed	1 (6%)	0	0	0	0	0	1 (17%)	1 (17%)	0	0	0	0	0
Body-mass index, kg/m^2^		24·2 (2·4)	24·6 (3·2)	24·4 (2·9)	26·2 (1·8)	22·0 (2·5)	25·2 (2·6)	24·6 (3·7)	23·3 (2·6)	25·7 (2·5)	28·7 (NC)	22·4 (2·8)	23·2 (2·9)	25·3 (2·7)
Height, cm		178·0 (6·8)	183·0 (10·4)	176·0 (7·7)	178·0 (6·7)	178·0 (3·9)	178·0 (7·8)	179·0 (6·8)	181·0 (4·9)	183·0 (9·3)	171·0 (NC)	175·0 (4·7)	176·0 (9·5)	181·0 (6·3)
Weight, kg		76·4 (10·4)	82·4 (14·9)	75·9 (10·6)	83·4 (9·7)	69·9 (9·9)	80·0 (10·4)	78·3 (12·7)	75·8 (7·6)	86·1 (9·4)	83·9 (NC)	68·8 (11·6)	72·7 (14·7)	83·4 (11·0)

Data are mean (SD) or n (%). 100% of participants in all groups were male. NC=not calculable.

A summary of adverse events reported after dosing with M5717 or placebo, including severity and relationship to the investigational product, is presented in table 2. Full lists of adverse events by system organ class and preferred term and adverse events considered related to study treatment are presented in the appendix (pp 2, 6). No adverse event in either the single ascending dose study or the volunteer infection study was classified as serious, and none resulted in study discontinuation. All adverse events related to M5717 were mild or moderate in severity, transient, and resolved without sequelae. No dose-related trends in vital signs, ECGs, or clinical laboratory parameters were detected in either the single ascending dose study or the volunteer infection study (data not shown).

**Table 2:** Adverse event summary by dose cohort

		**Single ascending dose study**										**Volunteer infection study**		
		Placebo (N=17)	50 mg (N=6)	100 mg (N=6)	200 mg (N=6)	400 mg (N=6)	600 mg (N=6)	1000 mg (N=6)	1250 mg (N=6)	1800 mg (N=6)	2100 mg (N=1)	150 mg (N=6)	400 mg (N=8)	800 mg (N=8)
Any adverse event		13 (76%)	6 (100%)	5 (83%)	3 (50%)	4 (67%)	3 (50%)	4 (67%)	5 (83%)	6 (100%)	1 (100%)	6 (100%)	8 (100%)	7 (88%)
	Related to study treatment	3 (18%)	0	0	0	1 (17%)	2 (33%)	1 (17%)	1 (17%)	6 (100%)	1 (100%)	0	1 (13%)	0
Moderate adverse event (grade 2)		1 (6%)	1 (17%)	1 (17%)	0	0	1 (17%)	0	1 (17%)	0	1 (100%)	2 (33%)	3 (38%)	2 (25%)
	Related to study treatment	0	0	0	0	0	0	0	1 (17%)	0	1 (100%)	0	0	0
Severe adverse event (grade 3)		0	1 (17%)	0	0	0	0	0	0	0	0	0	0	1 (13%)

Data are number of participants with at least one adverse event observed after dosing with M5717 or placebo (%). There were no serious adverse events, adverse events resulting in study discontinuation, grade 3 adverse events related to study treatment, or grade 4 adverse events reported in this study. The medical assessment of adverse event severity was done in accordance with the Qualitative Toxicity Scale^10^ (mild=grade 1, moderate=grade 2, severe=grade 3, potentially life threatening=grade 4).

In the single ascending dose study, 37 (76%) of 49 participants dosed with M5717 and 13 (76%) of 17 dosed with placebo experienced at least one adverse event (table 2). Adverse events related to M5717 or placebo were experienced by 12 (24%) of 49 participants dosed with M5717 and three (18%) of 17 dosed with placebo. No participant dosed with 50 mg, 100 mg, or 200 mg M5717 experienced an adverse event related to M5717. One (17%) of six participants in each of the 400 mg, 1000 mg, and 1250 mg M5717 dosing groups, and two (33%) of six in the 600 mg M5717 dosing group, experienced at least one adverse event related to M5717. All six participants dosed with 1800 mg M5717, as well as the single individual dosed with 2100 mg, experienced at least one adverse event related to M5717. The most commonly reported adverse events related to M5717 were headache (six participants), blurred vision (four participants), dizziness (three participants), and oral hypoesthesia (three participants; appendix p 6). Adverse events related to M5717 of abdominal discomfort, upper abdominal pain, dizziness, hot flush, and mental impairment were experienced only by participants dosed with 1800 mg M5717, and oral hypoesthesia and blurred vision were experienced only by participants dosed with 1800 mg or 2100 mg M5717 (appendix p 6). The onset of the nervous system disorder adverse events of blurred vision, oral hypoesthesia, and dizziness was around the time of t_max_, but we did not consider that there was a relationship with peak drug concentration, with these adverse events not occurring in some participants with high C_max_ (appendix p 7).

In the volunteer infection study, 21 (95%) of 22 participants experienced at least one adverse event after M5717 dosing. The majority of adverse events observed in the volunteer infection study were mild to moderate signs and symptoms that are frequently associated with malaria; the incidence of these adverse events declined over the course of approximately 7 days after M5717 dosing. Only one participant in the volunteer infection study (dosed with 400 mg M5717) experienced an adverse event related to M5717 (mild rhinorrhoea that resolved without sequelae; table 2; appendix p 6).

**Figure 2:**
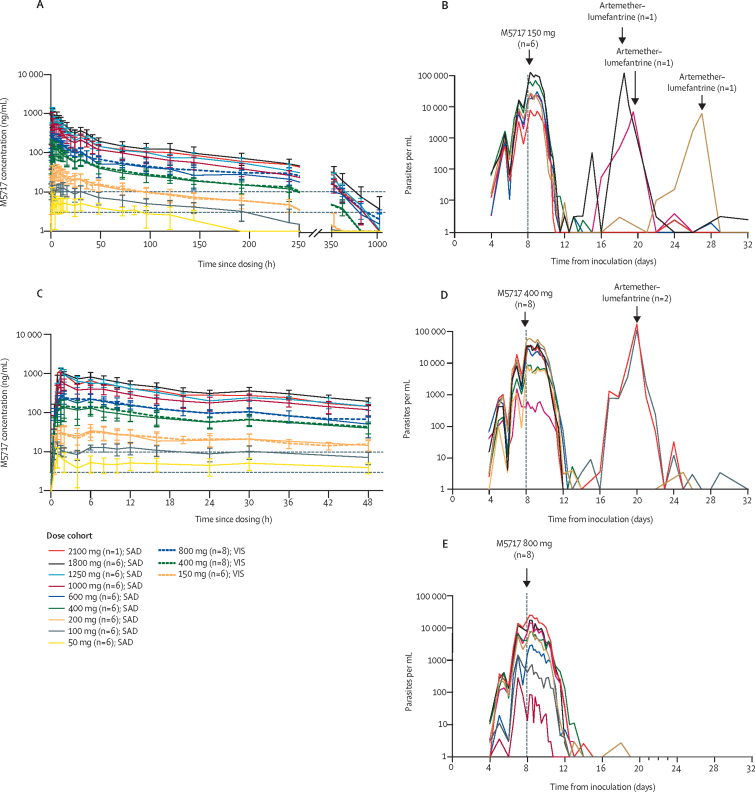
M5717 whole blood concentration–time profiles by dose cohort in the SAD and VIS (A and C) and individual-participant parasitaemia–time profiles in the VIS (B, D, and E) Plots represent mean values with SD (error bars) of the M5717 concentration of each dose cohort over the entire study (A) or over the first 48 h after dosing (C). Horizontal dotted lines indicate the minimum inhibitory concentration (3 ng/mL) and minimum parasiticidal concentration (10 ng/mL) estimated from preclinical efficacy studies in mice.^4^ For the purpose of graphing on a log_10_ logarithmic scale, timepoints at which M5717 could not be detected were substituted with a value of 1 ng/mL (the lower limit of quantitation). In the VIS, participants were inoculated intravenously with *Plasmodium falciparum*-infected erythrocytes and administered a single oral dose of 150 mg (B), 400 mg (D), or 800 mg (E) M5717 after 8 days (indicated by the vertical dashed line). Artemether–lumefantrine (six oral doses taken over 60 h; total dose of 480 mg artemether and 2·88 g lumefantrine) was administered in response to recrudescence of parasitaemia or 21 days after M5717 dosing if recrudescence was not observed. For the purpose of graphing on a log_10_ logarithmic scale, timepoints at which parasitaemia could not be detected were substituted with a value of 1 parasite per mL. SAD=single ascending dose study. VIS=volunteer infection study.

M5717 whole-blood concentrations peaked approximately 2 h after dosing across dose levels in both the single ascending dose study and the volunteer infection study, and this peak was followed by a biexponential decline (figure 2A). Multiple secondary peaks were observed around 6–10 h and 30 h after dosing (figure 2B). Dose-related increases in exposure were observed across the entire dose range evaluated in both the single ascending dose study and the volunteer infection study (table 3). Median t_max_ values ranged from 1 h to 7 h across dose groups in both the single ascending dose study and the volunteer infection study (table 3). Mean t_1/2_ values ranged from 106 h to 193 h and were lower at lower doses (≤200 mg), probably because M5717 concentrations fell below the LLOQ (1 ng/mL) earlier than in higher-dose groups for most participants in the lower-dose groups, leading to estimation inaccuracy. At doses above 200 mg, mean t_1/2_ values ranged from 146 h to 193 h. The earlier decline below the LLOQ might also have resulted in some underestimation of total exposure (AUC_0–∞_) and corresponding overestimation of CL/F at the lower doses. Mean Vz/F estimates were high, ranging from 5880 L to 9510 L across both the single ascending dose study and the volunteer infection study. The mean t_≥3 ng/mL_ and t_≥10 ng/mL_ increased with dose over the entire dose range in both the single ascending dose study and the volunteer infection study. Coefficients of variation ranged from 23·9% to 44·3% for AUC_0–∞_, and from 19·7% to 54·3% for C_max_, which is comparable to the variability observed for other substrates of CYP3A4.^19^ At doses of 150 mg and higher, M5717 concentrations were at least 3 ng/mL for at least 168 h in all participants, and at doses of 400 mg or higher, M5717 concentrations were 10 ng/mL or higher for at least 168 h in all participants. No discernible differences or trends were observed in pharmacokinetic parameters between the single ascending dose study and the volunteer infection study. The statistical assessment of dose proportionality of exposure across both the single ascending dose study and the volunteer infection study suggested a slightly greater-than-proportional increase for all AUC parameters and C_max_ (slope estimates of 1·16–1·32 for AUC parameters and 1·48 for C_max_; 90% CI above 1·00 for all parameters; data not shown in full). The apparent deviation from proportionality was small, and in the case of the AUC parameters, might be at least partially attributable to the underestimation at the lower doses.

**Table 3:** Pharmacokinetic parameters by dose cohort

	**Single ascending dose study**									**Volunteer infection study**		
	50 mg (N=6)	100 mg (N=6)	200 mg (N=6)	400 mg (N=6)	600 mg (N=6)	1000 mg (N=6)	1250 mg (N=6)	1800 mg (N=6)	2100 mg (N=1*)	150 mg (N=6)	400 mg (N=8)	800 mg (N=8)
AUC_0–∞_ (ng × h/mL)	997 (23·9%)†	1710 (44·3%)†	3500 (28·1%%)†	10 100 (24·9%)	17 500 (29·7%)	28 600 (29·8%)	37 800 (28·2%)	52 800 (32·4%)	43 000	3100 (41·0%)†	10 300 (40·4%)	20 000 (37·6%)
AUC_0–t_(ng × h/mL)	492 (53·7%)	1250 (50·0%)	2630 (32·6%)	9290 (25·5%)	15 800 (40·9%)	27 900 (29·7%)	37 000 (28·6%)	51 300 (33·0%)	42 200	2680 (44·9%)	9470 (42·9%)	19 200 (38·9%)
AUC_0–144h_(ng × h/mL)	475 (44·0%)	949 (30·3%)	1940 (28·9%)	5830 (29·6%)	9510 (22·4%)	18 400 (28·8%)	24 200 (24·0%)	32 200 (24·8%)	27 200	1930 (34·7%)	6260 (35·1%)	10 000 (28·4%)
C_max_ (ng/mL)	7·5 (54·3%)	14·9 (19·7%)	35·7 (41·3%)	146·0 (37·9%)	267·0 (25·4%)	642·0 (53·2%)	988·0 (40·3%)	1160·0 (22·7%)	1240·0	36·3 (37·0%)	174·0 (28·7%)	269·0 (48·2%)
t_max_ (h)	1·0 (1·0–1·0)	7·0 (1·0–12·0)	4·0 (0·5–8·0)	3·0 (1·5–6·0)	2·0 (1·0–6·0)	1·8 (1·5–10·0)	1·8 (1·5–8·0)	2·0 (1·5–6·0)	1·5	3·8 (1·0–12·0)	2·0 (1·0–8·0)	2·0 (1·5–6·0)
t_½_ (h)	133 (39·7%)†	133 (29·5%)†	145 (28·4%)†	155 (17·3%)	181 (25·6%)	169 (38·0%)	180 (16·7%)	181 (31·4%)	140	106 (19·4%)†	146 (14·9%)	193 (20·1%)
CL/F (L/h)	39·9 (23·9%)†	46·5 (44·3%)†	45·5 (28·1%)†	31·7 (24·9%)	27·4 (29·7%)	27·9 (29·8%)	26·3 (28·2%)	27·1 (32·4%)	38·8	38·4 (41·0%)†	31·1 (40·4%)	31·9 (37·6%)
Vz/F (L)	7640 (45·6%)†	8890 (18·3%)†	9510 (32·9%)†	7100 (24·3%)	7160 (24·1%)	6770 (38·7%)	6830 (22·5%)	7060 (24·8%)	7870	5880 (30·1%)†	6530 (28·7%)	8890 (26·8%)
t_≥3 ng/mL_(h)	80·1 (78·4%)	202·5 (37·4%)	336·2 (19·1%)	546·2 (14·2%)	649·6 (57·9%)	788·9 (22·2%)	846·2 (17·4%)	916·2 (21·2%)	867·0	299·7 (23·4%)	529·0 (23·9%)	804·4 (25·3%)
t_≥10 ng/mL_(h)	ND‡	11·0 (246·1%)	82·1 (48·3%)	278·6 (17·8%)	402·1 (35·6%)	463·2 (21·9%)	539·8 (19·3%)	679·1 (32·5%)	496·0	85·9 (56·7%)	273·1 (32·8%)	474·5 (31·1%)

Data are geometric means (coefficient of variation [%]) except t_max_, which is median (range). AUC_0–∞_=area under the concentration–time curve from time 0 h (dosing) extrapolated to infinity. AUC_0–t_=area under the concentration–time curve from time 0 h (dosing) to the last sampling time at which the concentration was at or above the lower limit of quantification. AUC_0–144h_=area under the concentration–time curve from time 0 h (dosing) to 144 h after dosing. C_max_=maximum observed concentration. t_max_=time to reach the maximum observed concentration. t_½_=apparent terminal half-life. CL/F=apparent total clearance. Vz/F=apparent volume of distribution. t_≥3 ng/mL_=time at or above the predicted minimum inhibitory concentration, calculated in mice to be 3 ng/mL. t_≥10 ng/mL_=time at or above the predicted minimum parasiticidal concentration, calculated in mice to be 10 ng/mL. ND=not determined.

* Descriptive statistics were not calculated since N=1.

† Pharmacokinetic diagnostics indicated non-reliability of the elimination rate constant estimate in ≥50% of participants, affecting related pharmacokinetic parameters.

‡ Concentrations were below 10 ng/mL at all timepoints in almost all participants.

The progression of parasitaemia after challenge in the volunteer infection study was somewhat variable between participants, particularly in the 800 mg cohort (figure 2C–E). The geometric mean parasitaemia at time of M5717 dosing was 18 931 parasites per mL (range 3362–58 958), 9912 parasites per mL (395–45 969), and 1857 parasites per mL (13–18 170) for the 150 mg, 400 mg, and 800 mg cohorts, respectively. Parasite clearance occurred in all participants following M5717 dosing, exhibiting a biphasic profile with parasitaemia decreasing slowly for 35–55 h after dosing, followed by rapid clearance. The breakpoint between the two phases and clearance kinetics for each phase were calculated (table 4; appendix p 8). The analysis of the 400 mg cohort (the first cohort enrolled in the volunteer infection study) was confounded by limited qPCR sampling undertaken across the timepoints when the second phase of clearance began, probably influencing the accuracy of the estimates. Two participants in this cohort were excluded from the cohort-specific estimates because the breakpoint between clearance phases for these individuals occurred later than in other participants in the cohort, resulting in insufficient datapoints to appropriately characterise the clearance slope of the second phase. Sampling timepoints for qPCR in the 150 mg and 800 mg cohorts were amended to enable estimation of clearance parameters with greater confidence. One participant in the 800 mg cohort was excluded from the cohort-specific estimates because parasitaemia was very low (13 parasites per mL at the time of dosing), resulting in difficulties in fitting the segmented regression. The reason for the low parasitaemia in this participant was unclear; the investigator confirmed that the participant did not meet the exclusion criteria associated with previous or concomitant medications and travel to malaria-endemic regions, and no errors in preparation of the malaria parasite inoculum were identified. The breakpoint between the two parasite clearance phases occurred later in the higher-dose cohorts than in the lowest-dose cohort. For all cohorts, parasite clearance was considerably faster during the second phase than in the first phase. There were differences in clearance kinetics between the 150 mg and 800 mg cohorts for both phases (95% CIs for PRR_48_ and half-life do not overlap; table 4). However, definitive comparisons between the 400 mg cohort and the other cohorts were limited by the aforementioned sampling timepoint issues.

**Table 4:** Parasite clearance parameters in the volunteer infection study

	**N**	**PRR_48_ (95% CI)**		**Parasite clearance half-life, h (95% CI)**		**Breakpoint between first and second phases, h (95% CI)**
		First phase	Second phase	First phase	Second phase	
150 mg	6	1·15 (0·59 to 2·25)	12 892 (3858 to 43 081)	231·1 (40·9 to not reached)	3·5 (3·1 to 4·0)	35·8 (31·3 to 40·3)
400 mg	6*	1·73 (1·27 to 2·37)	5127 (1006 to 26 132)	60·4 (38·6 to 138·6)	3·9 (3·3 to 4·8)	54·4 (47·2 to 61·6)
800 mg	7†	3·86 (2·90 to 5·13)	436 (179 to 1061)	24·7 (20·4 to 31·3)	5·5 (4·8 to 6·4)	55·7 (52·9 to 58·5)

Data represent cohort-specific estimates for each parameter calculated using random-effects meta-analysis.^18^ PRR_48_=parasite reduction ratio after treatment standardised over a 48 h period.

* Two participants were excluded from analyses in the 400 mg cohort since the breakpoint for these individuals occurred later than in other participants in the cohort and there were insufficient datapoints to appropriately characterise the second phase slope.

† One participant was excluded from analyses in the 800 mg cohort because parasitaemia was very low throughout the study, resulting in difficulties in fitting the segmented regression.

Recrudescence occurred in three (50%) of six participants in the 150 mg cohort (10, 11, and 19 days after M5717 dosing) and in two (25%) of eight in the 400 mg cohort (both at 12 days after M5717 dosing). No recrudescence was observed in the 800 mg cohort up to 21 days after dosing. All participants were treated with artemether–lumefantrine per protocol and were confirmed to be aparasitaemic by the end of the study. Sequencing of the full-length *P falciparum eEF2* gene was undertaken with recrudescent parasites from all five participants who experienced recrudescence (appendix p 10). Single-nucleotide polymorphisms were detected in four of the five recrudescent parasite populations (in two of three participants in the 150 mg cohort and both participants in the 400 mg cohort), resulting in amino acid substitutions at positions 134 (Glu to Gln), 183 (Ile to Met), 474 (Ser to Arg), and 754 (Pro to Ala) in *Pf*eEF2.

## Discussion

This study represents the first clinical investigation of the safety, pharmacokinetics, and antimalarial activity of M5717, the first plasmodium eEF2 inhibitor to reach clinical development. A major strength of this study is the combining of the single ascending dose study with the volunteer infection study in a first in-human study to accelerate early clinical evaluation of this compound. This approach enabled the rapid accrual of key safety and efficacy data.

Safety results indicated that M5717 is well tolerated when administered to healthy individuals as a single oral dose. No serious adverse events were observed during the study, and all adverse events considered related to M5717 were mild or moderate in severity and transient in nature. The incidence of M5717-related adverse events was low at doses up to and including 1250 mg (one [17%] of six participants dosed with 1250 mg M5717 in the single ascending dose study experienced at least one treatment-related adverse event). The side-effects of transient oral hypoesthesia and blurred vision (both mild or moderate in severity), which occurred among participants administered 1800 mg or 2100 mg M5717, were considered to be possible concentration-related parasympathetic effects. As nervous system disorder-related adverse events were not predicted from the animal toxicity studies, these adverse events were considered to constitute an unknown risk and, since multiples of the predicted therapeutic dose and exposure were reached, further dosing was suspended after dosing of the two sentinel participants in the 2100 mg cohort.

The pharmacokinetic profile of M5717 observed in humans confirms the potential for the compound to be developed as a new antimalarial. M5717 was rapidly absorbed following oral administration (maximum blood concentrations occurred between 1 h and 7 h after administration), and a very long half-life was observed (146–193 h at doses >200 mg), which is substantially longer than the half-life observed in preclinical animal studies following oral administration of 3–5 mg/kg (18–20 h).^4^ For all participants administered a dose of 400 mg or higher, M5717 blood concentrations remained above the minimum parasiticidal concentration estimated in the preclinical mouse efficacy model (10 ng/mL)^4^ for at least 7 days, which is longer than three parasite replication cycles. Preclinical studies indicated that M5717 is a substrate of cytochrome P450 3A4 (CYP3A4), with no active or toxic metabolites identified. Future drug–drug interaction studies will be needed to explore the effect of CYP3A4 inducers and inhibitors on M5717 pharmacokinetics.

Results obtained in the volunteer infection study indicate that M5717 has potent blood-stage antimalarial activity in humans. Single oral doses of 150, 400, or 800 mg M5717 were able to clear *P falciparum* parasitaemia initially. Doses of 150 mg or 400 mg were insufficient to prevent recrudescence in all participants, whereas no participant developed recurrent parasitaemia up to 21 days after dosing when administered 800 mg. The curative dose of M5717 will be estimated with population pharmacokinetic–pharmacodynamic modelling using data from this study, as previous studies have demonstrated a correlation between curative dose predictions from volunteer infection study data and clinical trial results.^13^, ^20^, ^21^ Importantly, the tolerability of single doses of M5717 above 800 mg was demonstrated in the single ascending dose study. Thus, a safety margin has been established in case of need for higher exposure in patients with higher parasitaemia burden and any differences in pharmacokinetics or pharmacodynamics in the target population for treatment.

The biphasic clearance profile observed following M5717 administration, with an initial period of slow clearance lasting 35–55 h (clearance half-life 24·7 h in the 800 mg cohort), followed by a rapid clearance phase (clearance half-life 5·5 h in the 800 mg cohort), was consistent with the antimalarial activity observed in vitro and in the mouse efficacy model, wherein a lag of 24–48 h was evident.^4^ Given that maximum blood concentrations of M5717 were reached 1–7 h after dosing, the apparent lag in parasite killing might reflect the mode of action of the compound in inhibiting protein synthesis. Alternatively, the lag might be due to the fact that the qPCR assay used to quantify parasitaemia measured all parasites in circulation (viable, damaged, or dead) and did not directly measure the rate of parasite killing. If the lag in parasite killing is indeed a true phenomenon, it suggests M5717 might need a partner drug that has a fast action with relatively long exposure if an indication for combined treatment of clinical disease is planned.

The importance of partnering M5717 with another antimalarial with a distinct mode and rapid onset of action is further highlighted by the finding that four of five parasite recrudescences had acquired genetic mutations in the target gene *PfeEF2* that are associated with resistance. All four unique amino acid substitutions occurred at or near mutations that have been previously identified in drug-resistant parasites obtained in vitro*.*^4^ In resistance-selection studies with *P falciparum* asexual blood-stage parasites, these mutations generally afforded five-fold to 40-fold increases in half-maximal growth inhibition concentration, with a minimum inoculum of resistance of approximately 10^7^ parasites, similar to the rate of resistance observed with the P-type ATPase 4 (PfATP4) inhibitor cipargamin^22^ and the dihydroorotate dehydrogenase (DHODH) inhibitor DSM265.^23^ In earlier studies, point mutations in *PfeEF2* resulted in a fitness cost to parasites, manifesting as reduced rates of parasite growth.^4^ Of note, in an in-vitro and in-vivo study using the mouse efficacy model wherein the hemozoin-formation inhibitor pyronaridine (a component of an approved artemisinin combination therapy) was partnered with M5717, pyronaridine prevented selection of M5717-resistant mutants, and M5717 did not negatively impact the rate of killing of the faster-acting pyronaridine.^24^ Furthermore, M5717 was previously shown to be highly effective against multiple *P falciparum* and *Plasmodium vivax* field isolates, as well as *P falciparum* culture-adapted lines resistant to chloroquine, pyrimethamine, and mefloquine.^4^

Although this study provides valuable first-in-human data on M5717, it has limitations. First, although women of non-childbearing potential were eligible for inclusion, none were enrolled. Therefore, it will be important to test for sex differences in safety and pharmacokinetics in future studies. Similarly, it will be important to investigate ethnic differences in these parameters, particularly in the target population in countries endemic for malaria. This study also did not investigate the effect of food on the pharmacokinetic profile of M5717. The variability in parasitaemia between cohorts at the time of dosing might have influenced the incidence of recrudescence observed, and the fact that definitive antimalarial treatment was initiated 21 days after dosing might have prevented observation of some late recrudescences. However, as noted already, pharmacokinetic–pharmacodynamic modelling is the preferred method for dose selection in early phase antimalarial drug development. The parasite clearance rate estimates are not expected to have been affected by variability in parasitaemia at the time of dosing because parasite clearance after treatment is considered a first-order process.^25^ Although volunteer infection studies have been shown to predict the activity of investigational antimalarials in studies in endemic populations,^12^, ^13^, ^20^, ^26^ further study of the pharmacodynamic effect of M5717 in such populations will be required to confirm that the selected dose is sufficient to effect cure.

In conclusion, this first-in-human study of the novel plasmodium eEF2 inhibitor M5717 supports its further clinical development as a new antimalarial. M5717 was well tolerated in healthy participants at doses that clear blood-stage *P falciparum,* and the long half-life of the compound indicates the potential for single-dose administration. The selection for resistance arising in these single-dose trials, which was also observed in a field trial on single dosing with the mitochondrial DHODH inhibitor DSM265,^20^ suggests that additional doses might be needed to mitigate the risk of resistance selection. Future studies to investigate combination therapies of M5717 with a fast-acting partner drug in treating clinical malaria are warranted, as are investigations into the use of M5717 for other indications, including chemoprotection, prophylaxis, and transmission blocking.

## Data sharing

Per company policy, following approval of a new product or a new indication for an approved product in both the EU and the USA, the healthcare business of Merck KGaA, Darmstadt, Germany, will share study protocols and study-level data and redacted clinical study reports from clinical trials in patients with qualified scientific and medical researchers, upon request, as necessary for conducting legitimate research. Further information on how to request data can be found on our website https://www.merckgroup.com/en/research/our-approach-to-research-and-development/healthcare/clinical-trials/commitment-responsible-data-sharing.html.

## Declaration of interests

ÖY, CO, AT, DB, XY, AK, and WMB are employed by the healthcare business of Merck KGaA, Darmstadt, Germany, the study sponsor. JSM (principal investigator) received funding from the healthcare business of Merck KGaA, Darmstadt, Germany, to perform the study. All other authors declare no competing interests.
