# Supplementary material for: Safety, pharmacokinetics, and antimalarial activity of the novel plasmodium eukaryotic translation elongation factor 2 inhibitor M5717: a first-in-human, randomised, placebo-controlled, double-blind, single ascending dose study and volunteer infection study
Source: Lancet Infect Dis. 2021 Dec;21(12):1713–24. doi: 10.1016/S1473-3099(21)00252-8 (PMC8612936; doi:10.1016/S1473-3099(21)00252-8)
Supplement: Supplementary appendix [file mmc1.pdf]

THE LANCET
Infectious Diseases

Supplementary appendix

This appendix formed part of the original submission and has been peer reviewed. We post it as supplied by the authors.

Supplement to: McCarthy JS, Yalkinoglu Ö, Odedra A, et al. Safety, pharmacokinetics, and antimalarial activity of the novel plasmodium eukaryotic translation elongation factor 2 inhibitor M5717: a first-in-human, randomised, placebo-controlled, double-blind, single ascending dose study and volunteer infection study. *Lancet Infect Dis* 2021; published online Oct 26. https://doi.org/10.1016/S1473-3099(21)00252-8.

Supplementary methods and results

Safety, pharmacokinetics and antimalarial activity of the novel *Plasmodium* eukaryotic translation elongation factor 2 inhibitor M5717: a first in human, randomised, placebo-controlled, double-blind, single ascending dose study and a volunteer infection study

James S. McCarthy, Özkan Yalkinoglu, Anand Odedra, Rebecca Webster, Claude Oeuvray, Aliona Tappert, Deon Bezuidenhout, Marla J. Giddins, Satish K. Dhingra, David A. Fidock, Louise Marquart, Lachlan Webb, Xiaoyan Yin, Akash Khandelwal, Wilhelmina M. Bagchus

Contents

Safety and tolerability results 2
Table S1. All adverse events by system organ class and preferred term (single ascending dose and volunteer infection studies) 2
Table S2. Adverse events related to M5717 or placebo by system organ class and preferred term (single ascending dose and volunteer infection studies) 6
Table S3. Occurrence of nervous system disorder adverse events in 1800 mg and 2100 mg dose cohorts and relationship to onset time, duration and M5717 blood concentration 7
Individual subject parasite clearance results in the volunteer infection study 8
Table S4. Individual subject parasite clearance parameters following M5717 administration 8
M5717 resistance assessment methods and results (volunteer infection study) 9
Table S5. Primers used for *PfeEF2* PCR amplification and sequencing 9
Table S6. Results of *PfeEF2* sequencing analysis 10
Measurement of M5717 in whole blood (single ascending dose and volunteer infection studies) 11
Table S7. Assay to determine M5717 concentrations in whole blood (diluted (1:1 with 100 mM citric acid) 11
General study information 12
Eligibility criteria for subject enrollment (single ascending dose and volunteer infection studies) 12
Blood sampling time points for M5717 concentration and parasitaemia measurements 15
Table S8. Key trial dates 16

Safety and tolerability results

Table S1. Adverse events by system organ class and preferred term (single ascending dose and volunteer infection studies)

	Single ascending dose study										Volunteer infection study		
**System Organ Class**<br>Preferred Term	**Placebo (n=17)**	**50 mg (n=6)**	**100 mg (n=6)**	**200 mg (n=6)**	**400 mg (n=6)**	**600 mg (n=6)**	**1000 mg (n=6)**	**1250 mg (n=6)**	**1800 mg (n=6)**	**2100 mg (n=1)**	**150 mg (n=6)**	**400 mg (n=8)**	**800 mg (n=8)**
	**Number of subjects with at least one adverse event observed after dosing with M5717 or placebo (%)**												
Any adverse event	13 (76.5)	6 (100.0)	5 (83.3)	3 (50.0)	4 (66.7)	3 (50.0)	4 (66.7)	5 (83.3)	6 (100.0)	1 (100.0)	6 (100.0)	8 (100.0)	7 (87.5)
**Blood and lymphatic system disorders**	**0 (0.0)**	**0 (0.0)**	**0 (0.0)**	**0 (0.0)**	**0 (0.0)**	**0 (0.0)**	**0 (0.0)**	**0 (0.0)**	**0 (0.0)**	**0 (0.0)**	**3 (50.0)**	**5 (62.5)**	**2 (25.0)**
Eosinophilia	0 (0.0)	0 (0.0)	0 (0.0)	0 (0.0)	0 (0.0)	0 (0.0)	0 (0.0)	0 (0.0)	0 (0.0)	0 (0.0)	1 (16.7)	0 (0.0)	0 (0.0)
Leukopenia	0 (0.0)	0 (0.0)	0 (0.0)	0 (0.0)	0 (0.0)	0 (0.0)	0 (0.0)	0 (0.0)	0 (0.0)	0 (0.0)	0 (0.0)	2 (25.0)	0 (0.0)
Lymphopenia	0 (0.0)	0 (0.0)	0 (0.0)	0 (0.0)	0 (0.0)	0 (0.0)	0 (0.0)	0 (0.0)	0 (0.0)	0 (0.0)	2 (33.3)	5 (62.5)	2 (25.0)
Neutropenia	0 (0.0)	0 (0.0)	0 (0.0)	0 (0.0)	0 (0.0)	0 (0.0)	0 (0.0)	0 (0.0)	0 (0.0)	0 (0.0)	0 (0.0)	2 (25.0)	1 (12.5)
**Cardiac disorders**	**0 (0.0)**	**1 (16.7)**	**0 (0.0)**	**0 (0.0)**	**0 (0.0)**	**0 (0.0)**	**0 (0.0)**	**0 (0.0)**	**0 (0.0)**	**0 (0.0)**	**1 (16.7)**	**6 (75.0)**	**3 (37.5)**
Tachycardia	0 (0.0)	1 (16.7)	0 (0.0)	0 (0.0)	0 (0.0)	0 (0.0)	0 (0.0)	0 (0.0)	0 (0.0)	0 (0.0)	1 (16.7)	6 (75.0)	3 (37.5)
**Gastrointestinal disorders**	**3 (17.6)**	**0 (0.0)**	**0 (0.0)**	**0 (0.0)**	**0 (0.0)**	**0 (0.0)**	**2 (33.3)**	**1 (16.7)**	**5 (83.3)**	**0 (0.0)**	**3 (50.0)**	**2 (25.0)**	**0 (0.0)**
Abdominal discomfort	0 (0.0)	0 (0.0)	0 (0.0)	0 (0.0)	0 (0.0)	0 (0.0)	0 (0.0)	0 (0.0)	2 (33.3)	0 (0.0)	0 (0.0)	2 (25.0)	0 (0.0)
Abdominal distension	0 (0.0)	0 (0.0)	0 (0.0)	0 (0.0)	0 (0.0)	0 (0.0)	2 (33.3)	0 (0.0)	1 (16.7)	0 (0.0)	0 (0.0)	0 (0.0)	0 (0.0)
Abdominal pain	1 (5.9)	0 (0.0)	0 (0.0)	0 (0.0)	0 (0.0)	0 (0.0)	0 (0.0)	0 (0.0)	0 (0.0)	0 (0.0)	1 (16.7)	0 (0.0)	0 (0.0)
Abdominal pain upper	0 (0.0)	0 (0.0)	0 (0.0)	0 (0.0)	0 (0.0)	0 (0.0)	0 (0.0)	0 (0.0)	1 (16.7)	0 (0.0)	0 (0.0)	0 (0.0)	0 (0.0)
Abdominal tenderness	0 (0.0)	0 (0.0)	0 (0.0)	0 (0.0)	0 (0.0)	0 (0.0)	0 (0.0)	0 (0.0)	0 (0.0)	0 (0.0)	0 (0.0)	2 (25.0)	0 (0.0)
Diarrhoea	1 (5.9)	0 (0.0)	0 (0.0)	0 (0.0)	0 (0.0)	0 (0.0)	0 (0.0)	0 (0.0)	2 (33.3)	0 (0.0)	0 (0.0)	0 (0.0)	0 (0.0)
Epigastric discomfort	0 (0.0)	0 (0.0)	0 (0.0)	0 (0.0)	0 (0.0)	0 (0.0)	0 (0.0)	1 (16.7)	0 (0.0)	0 (0.0)	0 (0.0)	0 (0.0)	0 (0.0)
Gingival pain	1 (5.9)	0 (0.0)	0 (0.0)	0 (0.0)	0 (0.0)	0 (0.0)	0 (0.0)	0 (0.0)	0 (0.0)	0 (0.0)	0 (0.0)	0 (0.0)	0 (0.0)
Mouth ulceration	1 (5.9)	0 (0.0)	0 (0.0)	0 (0.0)	0 (0.0)	0 (0.0)	0 (0.0)	0 (0.0)	0 (0.0)	0 (0.0)	0 (0.0)	0 (0.0)	0 (0.0)
Nausea	1 (5.9)	0 (0.0)	0 (0.0)	0 (0.0)	0 (0.0)	0 (0.0)	0 (0.0)	0 (0.0)	0 (0.0)	0 (0.0)	2 (33.3)	0 (0.0)	0 (0.0)
**General disorders and administration site conditions**	**1 (5.9)**	**2 (33.3)**	**0 (0.0)**	**0 (0.0)**	**0 (0.0)**	**0 (0.0)**	**0 (0.0)**	**1 (16.7)**	**1 (16.7)**	**0 (0.0)**	**2 (33.3)**	**1 (12.5)**	**1 (12.5)**
Catheter site bruise	0 (0.0)	1 (16.7)	0 (0.0)	0 (0.0)	0 (0.0)	0 (0.0)	0 (0.0)	0 (0.0)	0 (0.0)	0 (0.0)	0 (0.0)	0 (0.0)	0 (0.0)
Catheter site induration	0 (0.0)	0 (0.0)	0 (0.0)	0 (0.0)	0 (0.0)	0 (0.0)	0 (0.0)	0 (0.0)	1 (16.7)	0 (0.0)	0 (0.0)	0 (0.0)	0 (0.0)

	Single ascending dose study										Volunteer infection study		
**System Organ Class**<br>Preferred Term	**Placebo (n=17)**	**50 mg (n=6)**	**100 mg (n=6)**	**200 mg (n=6)**	**400 mg (n=6)**	**600 mg (n=6)**	**1000 mg (n=6)**	**1250 mg (n=6)**	**1800 mg (n=6)**	**2100 mg (n=1)**	**150 mg (n=6)**	**400 mg (n=8)**	**800 mg (n=8)**
Catheter site pain	0 (0.0)	0 (0.0)	0 (0.0)	0 (0.0)	0 (0.0)	0 (0.0)	0 (0.0)	0 (0.0)	1 (16.7)	0 (0.0)	0 (0.0)	0 (0.0)	0 (0.0)
Chills	0 (0.0)	1 (16.7)	0 (0.0)	0 (0.0)	0 (0.0)	0 (0.0)	0 (0.0)	0 (0.0)	0 (0.0)	0 (0.0)	0 (0.0)	0 (0.0)	1 (12.5)
Fatigue	1 (5.9)	0 (0.0)	0 (0.0)	0 (0.0)	0 (0.0)	0 (0.0)	0 (0.0)	0 (0.0)	0 (0.0)	0 (0.0)	0 (0.0)	1 (12.5)	0 (0.0)
Malaise	0 (0.0)	0 (0.0)	0 (0.0)	0 (0.0)	0 (0.0)	0 (0.0)	0 (0.0)	0 (0.0)	0 (0.0)	0 (0.0)	1 (16.7)	0 (0.0)	1 (12.5)
Pyrexia	0 (0.0)	0 (0.0)	0 (0.0)	0 (0.0)	0 (0.0)	0 (0.0)	0 (0.0)	0 (0.0)	0 (0.0)	0 (0.0)	2 (33.3)	0 (0.0)	1 (12.5)
Vessel puncture site bruise	0 (0.0)	0 (0.0)	0 (0.0)	0 (0.0)	0 (0.0)	0 (0.0)	0 (0.0)	1 (16.7)	0 (0.0)	0 (0.0)	0 (0.0)	0 (0.0)	0 (0.0)
**Infections and infestations**	**4 (23.5)**	**2 (33.3)**	**2 (33.3)**	**0 (0.0)**	**2 (33.3)**	**2 (33.3)**	**1 (16.7)**	**0 (0.0)**	**0 (0.0)**	**0 (0.0)**	**0 (0.0)**	**1 (12.5)**	**2 (25.0)**
Abscess limb	1 (5.9)	0 (0.0)	0 (0.0)	0 (0.0)	0 (0.0)	0 (0.0)	0 (0.0)	0 (0.0)	0 (0.0)	0 (0.0)	0 (0.0)	0 (0.0)	0 (0.0)
Folliculitis	0 (0.0)	0 (0.0)	0 (0.0)	0 (0.0)	1 (16.7)	0 (0.0)	0 (0.0)	0 (0.0)	0 (0.0)	0 (0.0)	0 (0.0)	0 (0.0)	0 (0.0)
Gastroenteritis	0 (0.0)	0 (0.0)	0 (0.0)	0 (0.0)	0 (0.0)	0 (0.0)	0 (0.0)	0 (0.0)	0 (0.0)	0 (0.0)	0 (0.0)	0 (0.0)	1 (12.5)
Hordeolum	1 (5.9)	0 (0.0)	0 (0.0)	0 (0.0)	0 (0.0)	0 (0.0)	0 (0.0)	0 (0.0)	0 (0.0)	0 (0.0)	0 (0.0)	0 (0.0)	0 (0.0)
Rhinitis	0 (0.0)	0 (0.0)	0 (0.0)	0 (0.0)	0 (0.0)	1 (16.7)	0 (0.0)	0 (0.0)	0 (0.0)	0 (0.0)	0 (0.0)	0 (0.0)	0 (0.0)
Upper respiratory tract infection	2 (11.8)	2 (33.3)	2 (33.3)	0 (0.0)	1 (16.7)	1 (16.7)	1 (16.7)	0 (0.0)	0 (0.0)	0 (0.0)	0 (0.0)	0 (0.0)	1 (12.5)
Viral upper respiratory tract infection	0 (0.0)	0 (0.0)	0 (0.0)	0 (0.0)	0 (0.0)	0 (0.0)	0 (0.0)	0 (0.0)	0 (0.0)	0 (0.0)	0 (0.0)	1 (12.5)	0 (0.0)
**Injury, poisoning and procedural complications**	**6 (35.3)**	**1 (16.7)**	**1 (16.7)**	**2 (33.3)**	**1 (16.7)**	**1 (16.7)**	**0 (0.0)**	**3 (50.0)**	**0 (0.0)**	**0 (0.0)**	**1 (16.7)**	**1 (12.5)**	**4 (50.0)**
Arthropod bite	0 (0.0)	0 (0.0)	0 (0.0)	0 (0.0)	0 (0.0)	0 (0.0)	0 (0.0)	0 (0.0)	0 (0.0)	0 (0.0)	0 (0.0)	1 (12.5)	1 (12.5)
Contusion	1 (5.9)	1 (16.7)	0 (0.0)	1 (16.7)	1 (16.7)	0 (0.0)	0 (0.0)	1 (16.7)	0 (0.0)	0 (0.0)	0 (0.0)	0 (0.0)	0 (0.0)
Skin abrasion	0 (0.0)	0 (0.0)	0 (0.0)	0 (0.0)	0 (0.0)	1 (16.7)	0 (0.0)	0 (0.0)	0 (0.0)	0 (0.0)	0 (0.0)	0 (0.0)	0 (0.0)
Sunburn	3 (17.6)	0 (0.0)	1 (16.7)	1 (16.7)	0 (0.0)	0 (0.0)	0 (0.0)	1 (16.7)	0 (0.0)	0 (0.0)	0 (0.0)	0 (0.0)	3 (37.5)
Thermal burn	1 (5.9)	0 (0.0)	0 (0.0)	0 (0.0)	0 (0.0)	0 (0.0)	0 (0.0)	1 (16.7)	0 (0.0)	0 (0.0)	0 (0.0)	0 (0.0)	0 (0.0)
Vascular access site bruising	1 (5.9)	0 (0.0)	0 (0.0)	0 (0.0)	0 (0.0)	0 (0.0)	0 (0.0)	0 (0.0)	0 (0.0)	0 (0.0)	0 (0.0)	0 (0.0)	0 (0.0)
Wound	0 (0.0)	0 (0.0)	0 (0.0)	0 (0.0)	0 (0.0)	0 (0.0)	0 (0.0)	0 (0.0)	0 (0.0)	0 (0.0)	1 (16.7)	0 (0.0)	0 (0.0)
**Investigations**	**0 (0.0)**	**3 (50.0)**	**0 (0.0)**	**0 (0.0)**	**0 (0.0)**	**0 (0.0)**	**0 (0.0)**	**0 (0.0)**	**0 (0.0)**	**0 (0.0)**	**3 (50.0)**	**0 (0.0)**	**1 (12.5)**
Alanine aminotransferase increased	0 (0.0)	1 (16.7)	0 (0.0)	0 (0.0)	0 (0.0)	0 (0.0)	0 (0.0)	0 (0.0)	0 (0.0)	0 (0.0)	2 (33.3)	0 (0.0)	0 (0.0)
Aspartate aminotransferase increased	0 (0.0)	1 (16.7)	0 (0.0)	0 (0.0)	0 (0.0)	0 (0.0)	0 (0.0)	0 (0.0)	0 (0.0)	0 (0.0)	2 (33.3)	0 (0.0)	0 (0.0)
Blood corticotrophin decreased	0 (0.0)	1 (16.7)	0 (0.0)	0 (0.0)	0 (0.0)	0 (0.0)	0 (0.0)	0 (0.0)	0 (0.0)	0 (0.0)	0 (0.0)	0 (0.0)	0 (0.0)
Blood creatine phosphokinase increased	0 (0.0)	1 (16.7)	0 (0.0)	0 (0.0)	0 (0.0)	0 (0.0)	0 (0.0)	0 (0.0)	0 (0.0)	0 (0.0)	1 (16.7)	0 (0.0)	1 (12.5)

	Single ascending dose study										Volunteer infection study		
**System Organ Class** Preferred Term	**Placebo (n=17)**	**50 mg (n=6)**	**100 mg (n=6)**	**200 mg (n=6)**	**400 mg (n=6)**	**600 mg (n=6)**	**1000 mg (n=6)**	**1250 mg (n=6)**	**1800 mg (n=6)**	**2100 mg (n=1)**	**150 mg (n=6)**	**400 mg (n=8)**	**800 mg (n=8)**
Lymph node palpable	0 (0.0)	1 (16.7)	0 (0.0)	0 (0.0)	0 (0.0)	0 (0.0)	0 (0.0)	0 (0.0)	0 (0.0)	0 (0.0)	0 (0.0)	0 (0.0)	0 (0.0)
**Metabolism and nutrition disorders**	**0 (0.0)**	**0 (0.0)**	**0 (0.0)**	**0 (0.0)**	**0 (0.0)**	**0 (0.0)**	**0 (0.0)**	**0 (0.0)**	**0 (0.0)**	**0 (0.0)**	**1 (16.7)**	**0 (0.0)**	**0 (0.0)**
Decreased appetite	0 (0.0)	0 (0.0)	0 (0.0)	0 (0.0)	0 (0.0)	0 (0.0)	0 (0.0)	0 (0.0)	0 (0.0)	0 (0.0)	1 (16.7)	0 (0.0)	0 (0.0)
**Musculoskeletal and connective tissue disorders**	**2 (11.8)**	**1 (16.7)**	**0 (0.0)**	**0 (0.0)**	**0 (0.0)**	**0 (0.0)**	**1 (16.7)**	**0 (0.0)**	**0 (0.0)**	**0 (0.0)**	**4 (66.7)**	**2 (25.0)**	**2 (25.0)**
Arthralgia	1 (5.9)	0 (0.0)	0 (0.0)	0 (0.0)	0 (0.0)	0 (0.0)	1 (16.7)	0 (0.0)	0 (0.0)	0 (0.0)	1 (16.7)	0 (0.0)	1 (12.5)
Back pain	0 (0.0)	0 (0.0)	0 (0.0)	0 (0.0)	0 (0.0)	0 (0.0)	0 (0.0)	0 (0.0)	0 (0.0)	0 (0.0)	0 (0.0)	1 (12.5)	0 (0.0)
Groin pain	0 (0.0)	0 (0.0)	0 (0.0)	0 (0.0)	0 (0.0)	0 (0.0)	0 (0.0)	0 (0.0)	0 (0.0)	0 (0.0)	0 (0.0)	0 (0.0)	1 (12.5)
Musculoskeletal stiffness	0 (0.0)	0 (0.0)	0 (0.0)	0 (0.0)	0 (0.0)	0 (0.0)	0 (0.0)	0 (0.0)	0 (0.0)	0 (0.0)	0 (0.0)	0 (0.0)	1 (12.5)
Myalgia	0 (0.0)	0 (0.0)	0 (0.0)	0 (0.0)	0 (0.0)	0 (0.0)	0 (0.0)	0 (0.0)	0 (0.0)	0 (0.0)	4 (66.7)	2 (25.0)	1 (12.5)
Neck pain	1 (5.9)	1 (16.7)	0 (0.0)	0 (0.0)	0 (0.0)	0 (0.0)	0 (0.0)	0 (0.0)	0 (0.0)	0 (0.0)	0 (0.0)	0 (0.0)	0 (0.0)
**Nervous system disorders**	**4 (23.5)**	**0 (0.0)**	**2 (33.3)**	**1 (16.7)**	**3 (50.0)**	**2 (33.3)**	**2 (33.3)**	**1 (16.7)**	**5 (83.3)**	**1 (100.0)**	**4 (66.7)**	**5 (62.5)**	**4 (50.0)**
Dizziness	1 (5.9)	0 (0.0)	0 (0.0)	0 (0.0)	0 (0.0)	0 (0.0)	1 (16.7)	0 (0.0)	3 (50.0)	0 (0.0)	1 (16.7)	0 (0.0)	0 (0.0)
Headache	3 (17.6)	0 (0.0)	2 (33.3)	1 (16.7)	3 (50.0)	2 (33.3)	1 (16.7)	0 (0.0)	4 (66.7)	0 (0.0)	3 (50.0)	5 (62.5)	4 (50.0)
Hypoaesthesia oral	0 (0.0)	0 (0.0)	0 (0.0)	0 (0.0)	0 (0.0)	0 (0.0)	0 (0.0)	0 (0.0)	2 (33.3)	1 (100.0)	0 (0.0)	0 (0.0)	0 (0.0)
Lethargy	0 (0.0)	0 (0.0)	0 (0.0)	0 (0.0)	0 (0.0)	0 (0.0)	0 (0.0)	0 (0.0)	0 (0.0)	0 (0.0)	2 (33.3)	0 (0.0)	0 (0.0)
Mental impairment	0 (0.0)	0 (0.0)	0 (0.0)	0 (0.0)	0 (0.0)	0 (0.0)	0 (0.0)	0 (0.0)	1 (16.7)	0 (0.0)	0 (0.0)	0 (0.0)	0 (0.0)
Paraesthesia	1 (5.9)	0 (0.0)	0 (0.0)	0 (0.0)	0 (0.0)	0 (0.0)	0 (0.0)	0 (0.0)	0 (0.0)	0 (0.0)	0 (0.0)	0 (0.0)	0 (0.0)
Presyncope	0 (0.0)	0 (0.0)	0 (0.0)	0 (0.0)	0 (0.0)	1 (16.7)	0 (0.0)	0 (0.0)	0 (0.0)	0 (0.0)	0 (0.0)	0 (0.0)	0 (0.0)
Sensory disturbance	0 (0.0)	0 (0.0)	0 (0.0)	0 (0.0)	0 (0.0)	0 (0.0)	0 (0.0)	1 (16.7)	0 (0.0)	0 (0.0)	0 (0.0)	0 (0.0)	0 (0.0)
Vision blurred	0 (0.0)	0 (0.0)	0 (0.0)	0 (0.0)	0 (0.0)	0 (0.0)	0 (0.0)	0 (0.0)	3 (50.0)	1 (100.0)	0 (0.0)	0 (0.0)	0 (0.0)
**Respiratory, thoracic and mediastinal disorders**	**1 (5.9)**	**3 (50.0)**	**1 (16.7)**	**0 (0.0)**	**0 (0.0)**	**0 (0.0)**	**1 (16.7)**	**0 (0.0)**	**0 (0.0)**	**0 (0.0)**	**1 (16.7)**	**2 (25.0)**	**0 (0.0)**
Dysphonia	0 (0.0)	1 (16.7)	0 (0.0)	0 (0.0)	0 (0.0)	0 (0.0)	0 (0.0)	0 (0.0)	0 (0.0)	0 (0.0)	0 (0.0)	0 (0.0)	0 (0.0)
Epistaxis	0 (0.0)	0 (0.0)	1 (16.7)	0 (0.0)	0 (0.0)	0 (0.0)	0 (0.0)	0 (0.0)	0 (0.0)	0 (0.0)	0 (0.0)	0 (0.0)	0 (0.0)
Oropharyngeal pain	0 (0.0)	3 (50.0)	0 (0.0)	0 (0.0)	0 (0.0)	0 (0.0)	0 (0.0)	0 (0.0)	0 (0.0)	0 (0.0)	1 (16.7)	1 (12.5)	0 (0.0)
Rhinorrhoea	1 (5.9)	1 (16.7)	0 (0.0)	0 (0.0)	0 (0.0)	0 (0.0)	1 (16.7)	0 (0.0)	0 (0.0)	0 (0.0)	0 (0.0)	2 (25.0)	0 (0.0)
Sneezing	0 (0.0)	0 (0.0)	0 (0.0)	0 (0.0)	0 (0.0)	0 (0.0)	0 (0.0)	0 (0.0)	0 (0.0)	0 (0.0)	0 (0.0)	1 (12.5)	0 (0.0)

	Single ascending dose study										Volunteer infection study		
**System Organ Class**<br>Preferred Term	**Placebo (n=17)**	**50 mg (n=6)**	**100 mg (n=6)**	**200 mg (n=6)**	**400 mg (n=6)**	**600 mg (n=6)**	**1000 mg (n=6)**	**1250 mg (n=6)**	**1800 mg (n=6)**	**2100 mg (n=1)**	**150 mg (n=6)**	**400 mg (n=8)**	**800 mg (n=8)**
**Skin and subcutaneous tissue disorders**	**1 (5.9)**	**0 (0.0)**	**1 (16.7)**	**0 (0.0)**	**0 (0.0)**	**0 (0.0)**	**1 (16.7)**	**2 (33.3)**	**0 (0.0)**	**0 (0.0)**	**1 (16.7)**	**1 (12.5)**	**1 (12.5)**
Dermatitis contact	1 (5.9)	0 (0.0)	0 (0.0)	0 (0.0)	0 (0.0)	0 (0.0)	0 (0.0)	0 (0.0)	0 (0.0)	0 (0.0)	0 (0.0)	0 (0.0)	0 (0.0)
Erythema	0 (0.0)	0 (0.0)	0 (0.0)	0 (0.0)	0 (0.0)	0 (0.0)	1 (16.7)	1 (16.7)	0 (0.0)	0 (0.0)	0 (0.0)	0 (0.0)	0 (0.0)
Hyperhidrosis	0 (0.0)	0 (0.0)	0 (0.0)	0 (0.0)	0 (0.0)	0 (0.0)	0 (0.0)	0 (0.0)	0 (0.0)	0 (0.0)	1 (16.7)	0 (0.0)	0 (0.0)
Pruritus	0 (0.0)	0 (0.0)	1 (16.7)	0 (0.0)	0 (0.0)	0 (0.0)	0 (0.0)	0 (0.0)	0 (0.0)	0 (0.0)	0 (0.0)	0 (0.0)	0 (0.0)
Rash	0 (0.0)	0 (0.0)	0 (0.0)	0 (0.0)	0 (0.0)	0 (0.0)	0 (0.0)	1 (16.7)	0 (0.0)	0 (0.0)	0 (0.0)	0 (0.0)	0 (0.0)
Rash erythematous	0 (0.0)	0 (0.0)	0 (0.0)	0 (0.0)	0 (0.0)	0 (0.0)	0 (0.0)	0 (0.0)	0 (0.0)	0 (0.0)	0 (0.0)	0 (0.0)	1 (12.5)
Seborrhoeic dermatitis	0 (0.0)	0 (0.0)	0 (0.0)	0 (0.0)	0 (0.0)	0 (0.0)	0 (0.0)	0 (0.0)	0 (0.0)	0 (0.0)	0 (0.0)	1 (12.5)	0 (0.0)
**Vascular disorders**	**0 (0.0)**	**0 (0.0)**	**1 (16.7)**	**0 (0.0)**	**0 (0.0)**	**1 (16.7)**	**0 (0.0)**	**1 (16.7)**	**1 (16.7)**	**0 (0.0)**	**0 (0.0)**	**1 (12.5)**	**0 (0.0)**
Flushing	0 (0.0)	0 (0.0)	0 (0.0)	0 (0.0)	0 (0.0)	1 (16.7)	0 (0.0)	0 (0.0)	0 (0.0)	0 (0.0)	0 (0.0)	0 (0.0)	0 (0.0)
Hot flush	0 (0.0)	0 (0.0)	0 (0.0)	0 (0.0)	0 (0.0)	0 (0.0)	0 (0.0)	0 (0.0)	1 (16.7)	0 (0.0)	0 (0.0)	1 (12.5)	0 (0.0)
Orthostatic hypotension	0 (0.0)	0 (0.0)	0 (0.0)	0 (0.0)	0 (0.0)	0 (0.0)	0 (0.0)	1 (16.7)	0 (0.0)	0 (0.0)	0 (0.0)	0 (0.0)	0 (0.0)
Thrombophlebitis superficial	0 (0.0)	0 (0.0)	1 (16.7)	0 (0.0)	0 (0.0)	0 (0.0)	0 (0.0)	0 (0.0)	0 (0.0)	0 (0.0)	0 (0.0)	0 (0.0)	0 (0.0)

Table S2. Adverse events related to M5717 or placebo by system organ class and preferred term (single ascending dose and volunteer infection studies)

	Single ascending dose study										Volunteer infection study		
**System Organ Class**<br>Preferred Term	**Placebo (n=17)**	**50 mg (n=6)**	**100 mg (n=6)**	**200 mg (n=6)**	**400 mg (n=6)**	**600 mg (n=6)**	**1000 mg (n=6)**	**1250 mg (n=6)**	**1800 mg (n=6)**	**2100 mg (n=1)**	**150 mg (n=6)**	**400 mg (n=8)**	**800 mg (n=8)**
	**Number of subjects with at least one adverse event observed after dosing with M5717 or placebo (%)**												
Any adverse event related to M5717/placebo	3 (17.6)	0 (0.0)	0 (0.0)	0 (0.0)	1 (16.7)	2 (33.3)	1 (16.7)	1 (16.7)	6 (100.0)	1 (100.0)	0 (0.0)	1 (12.5)	0 (0.0)
**Gastrointestinal disorders**	**2 (11.8)**	**0 (0.0)**	**0 (0.0)**	**0 (0.0)**	**0 (0.0)**	**0 (0.0)**	**1 (16.7)**	**0 (0.0)**	**5 (83.3)**	**0 (0.0)**	**0 (0.0)**	**0 (0.0)**	**0 (0.0)**
Abdominal discomfort	0 (0.0)	0 (0.0)	0 (0.0)	0 (0.0)	0 (0.0)	0 (0.0)	0 (0.0)	0 (0.0)	2 (33.3)	0 (0.0)	0 (0.0)	0 (0.0)	0 (0.0)
Abdominal distension	0 (0.0)	0 (0.0)	0 (0.0)	0 (0.0)	0 (0.0)	0 (0.0)	1 (16.7)	0 (0.0)	1 (16.7)	0 (0.0)	0 (0.0)	0 (0.0)	0 (0.0)
Abdominal pain	1 (5.9)	0 (0.0)	0 (0.0)	0 (0.0)	0 (0.0)	0 (0.0)	0 (0.0)	0 (0.0)	0 (0.0)	0 (0.0)	0 (0.0)	0 (0.0)	0 (0.0)
Abdominal pain upper	0 (0.0)	0 (0.0)	0 (0.0)	0 (0.0)	0 (0.0)	0 (0.0)	0 (0.0)	0 (0.0)	1 (16.7)	0 (0.0)	0 (0.0)	0 (0.0)	0 (0.0)
Diarrhoea	1 (5.9)	0 (0.0)	0 (0.0)	0 (0.0)	0 (0.0)	0 (0.0)	0 (0.0)	0 (0.0)	2 (33.3)	0 (0.0)	0 (0.0)	0 (0.0)	0 (0.0)
Gingival pain	1 (5.9)	0 (0.0)	0 (0.0)	0 (0.0)	0 (0.0)	0 (0.0)	0 (0.0)	0 (0.0)	0 (0.0)	0 (0.0)	0 (0.0)	0 (0.0)	0 (0.0)
Nausea	1 (5.9)	0 (0.0)	0 (0.0)	0 (0.0)	0 (0.0)	0 (0.0)	0 (0.0)	0 (0.0)	0 (0.0)	0 (0.0)	0 (0.0)	0 (0.0)	0 (0.0)
**Nervous system disorders**	**1 (5.9)**	**0 (0.0)**	**0 (0.0)**	**0 (0.0)**	**1 (16.7)**	**1 (16.7)**	**0 (0.0)**	**0 (0.0)**	**5 (83.3)**	**1 (100.0)**	**0 (0.0)**	**0 (0.0)**	**0 (0.0)**
Dizziness	0 (0.0)	0 (0.0)	0 (0.0)	0 (0.0)	0 (0.0)	0 (0.0)	0 (0.0)	0 (0.0)	3 (50.0)	0 (0.0)	0 (0.0)	0 (0.0)	0 (0.0)
Headache	1 (5.9)	0 (0.0)	0 (0.0)	0 (0.0)	1 (16.7)	1 (16.7)	0 (0.0)	0 (0.0)	4 (66.7)	0 (0.0)	0 (0.0)	0 (0.0)	0 (0.0)
Hypoaesthesia oral	0 (0.0)	0 (0.0)	0 (0.0)	0 (0.0)	0 (0.0)	0 (0.0)	0 (0.0)	0 (0.0)	2 (33.3)	1 (100.0)	0 (0.0)	0 (0.0)	0 (0.0)
Mental impairment	0 (0.0)	0 (0.0)	0 (0.0)	0 (0.0)	0 (0.0)	0 (0.0)	0 (0.0)	0 (0.0)	1 (16.7)	0 (0.0)	0 (0.0)	0 (0.0)	0 (0.0)
Vision blurred	0 (0.0)	0 (0.0)	0 (0.0)	0 (0.0)	0 (0.0)	0 (0.0)	0 (0.0)	0 (0.0)	3 (50.0)	1 (100.0)	0 (0.0)	0 (0.0)	0 (0.0)
**Respiratory, thoracic and mediastinal disorders**	**1 (5.9)**	**0 (0.0)**	**0 (0.0)**	**0 (0.0)**	**0 (0.0)**	**0 (0.0)**	**0 (0.0)**	**0 (0.0)**	**0 (0.0)**	**0 (0.0)**	**0 (0.0)**	**1 (12.5)**	**0 (0.0)**
Rhinorrhoea	1 (5.9)	0 (0.0)	0 (0.0)	0 (0.0)	0 (0.0)	0 (0.0)	0 (0.0)	0 (0.0)	0 (0.0)	0 (0.0)	0 (0.0)	1 (12.5)	0 (0.0)
**Vascular disorders**	**0 (0.0)**	**0 (0.0)**	**0 (0.0)**	**0 (0.0)**	**0 (0.0)**	**1 (16.7)**	**0 (0.0)**	**1 (16.7)**	**1 (16.7)**	**0 (0.0)**	**0 (0.0)**	**0 (0.0)**	**0 (0.0)**
Flushing	0 (0.0)	0 (0.0)	0 (0.0)	0 (0.0)	0 (0.0)	1 (16.7)	0 (0.0)	0 (0.0)	0 (0.0)	0 (0.0)	0 (0.0)	0 (0.0)	0 (0.0)
Hot flush	0 (0.0)	0 (0.0)	0 (0.0)	0 (0.0)	0 (0.0)	0 (0.0)	0 (0.0)	0 (0.0)	1 (16.7)	0 (0.0)	0 (0.0)	0 (0.0)	0 (0.0)
Orthostatic hypotension	0 (0.0)	0 (0.0)	0 (0.0)	0 (0.0)	0 (0.0)	0 (0.0)	0 (0.0)	1 (16.7)	0 (0.0)	0 (0.0)	0 (0.0)	0 (0.0)	0 (0.0)

Table S3. Occurrence of nervous system disorder adverse events in 1800 mg and 2100 mg dose cohorts and relationship to onset time, duration and M5717 blood concentration

Adverse event	Subject identifier	Dose (mg)	Interval from dosing to onset (h)	Duration (h)	M5717 concentration at time point closest to onset of AE (ng/mL [h])	T_{max} (h)	C_{max} (ng/mL)
Blurred vision	202	1800	1.2	45.0	394 [1]	2.0	967
	204	1800	1.0	13.0	1180 [1]	1.5	1750
	208	1800	1.8	44.8	439 [1.5]	2.0	951
	211	2100	1.0	18.5	1040 [1]	1.5	1240
Oral hypoesthesia	202	1800	1.4	44.8	774 [1.5]	2.0	967
	204	1800	1.1	2.9	1180 [1]	1.5	1750
	211	2100	1.3	4.2	1240 [1.5]	1.5	1240
Dizziness	192	1800	1.1	11.3	812 [1]	6.0	1220
	197	1800	1.9	6.3	1190 [2]	2.0	1190
	208	1800	1.8	10.8	439 [1.5]	2.0	951
None of the above AEs							
	210	*1800*				*1.5*	*1070*
	162	*1250*				*1.5*	*1410*
	175	*1250*				*1.5*	*1560*
	186	*1250*				*2.0*	*1140*
	144	*1000*				*1.5*	*1270*

Individual subject parasite clearance results in the volunteer infection study

Table S4. Individual subject parasite clearance parameters following M5717 administration

Subject ID	PRR_{48} (95%CI)		Parasite Clearance Half-Life [h (95%CI)]		Break point [h (95%CI)]
	First phase	Second phase	First phase	Second phase	
**Cohort 1 (400 mg M5717)**					
Subject 1	1.23 (0.59 - 2.55)	1047 (477 - 2299)	162.2 (35.6 - ∞)	4.8 (4.3 - 5.4)	43.2 (39.6 - 46.7)
Subject 2	1.86 (0.78 - 4.46)	62492 (17033 - 229171)	53.5 (22.3 - ∞)	3.0 (2.7 - 3.4)	55.7 (51.9 - 59.5)
Subject 3	2.95 (1.16 - 7.52)	50936 (12666 - 204739)	30.7 (16.5 - 225.5)	3.1 (2.7 - 3.5)	54.7 (50.4 - 59.1)
Subject 4	1.38 (0.11 - 18.10)	131 (31 - 558)	102.7 (11.5 - ∞)	6.8 (5.3 - 9.7)	48.0 (26.2 - 69.8)
Subject 5[a]	2.56 (1.48 - 4.42)	8283998279 (99046698 - 692851242294)	35.4 (22.4 - 84.2)	1.5 (1.2 - 1.8)	80.0 (77.6 - 82.3)
Subject 6	2.35 (1.15 - 4.78)	23464 (5695 - 96667)	39.0 (21.3 - 231.2)	3.3 (2.9 - 3.9)	66.7 (62.0 - 71.3)
Subject 7[a]	2.57 (1.43 - 4.62)	5614098 (49302 - 639852690)	35.2 (21.8 - 92.1)	2.1 (1.6 - 3.1)	81.1 (77.7 - 84.6)
Subject 8	1.16 (0.51 - 2.60)	1947 (1021 - 3712)	227.4 (34.8 - ∞)	4.4 (4.1 - 4.8)	54.6 (50.0 - 59.2)
**Cohort 2 (150 mg M5717)**					
Subject 1	1.70 (0.31 - 9.28)	13982 (5017 - 38969)	62.6 (14.9 - ∞)	3.5 (3.2 - 3.9)	34.3 (30.6 - 38.0)
Subject 2	2.19 (0.03 - 138.29)	4422 (1382 - 14147)	42.4 (6.8 - ∞)	4.0 (3.5 - 4.6)	28.6 (20.5 - 36.6)
Subject 3	2.71 (0.40 - 18.56)	3648211 (137379 - 96881309)	33.3 (11.4 - ∞)	2.2 (1.8 - 2.8)	46.0 (41.6 - 50.5)
Subject 4	1.27 (0.02 - 79.61)	1764 (579 - 5377)	137.5 (7.6 - ∞)	4.5 (3.9 - 5.2)	31.9 (22.0 - 41.9)
Subject 5	0.50 (0.14 - 1.77)	5076 (1944 - 13257)	-48.6 (58.3 - ∞)	3.9 (3.5 - 4.4)	33.5 (30.8 - 36.2)
Subject 6	1.86 (0.30 - 11.63)	34050 (9158 - 126590)	53.4 (13.6 - ∞)	3.2 (2.8 - 3.7)	37.1 (32.6 - 41.6)
**Cohort 3 (800 mg M5717)**					
Subject 1	2.89 (1.18 - 7.08)	184 (65 - 520)	31.3 (17.0 - 198.6)	6.4 (5.3 – 8.0)	58.8 (49.8 - 67.8)
Subject 2	5.54 (3.58 - 8.57)	2086 (1011 - 4304)	19.4 (15.5 - 26.1)	4.4 (4.0 - 4.8)	55.7 (52.5 - 59.0)
Subject 3	2.83 (1.34 – 6.00)	610 (379 - 983)	32.0 (18.6 - 114.3)	5.2 (4.8 - 5.6)	51.3 (46.5 - 56.0)
Subject 4	2.49 (1.24 – 5.00)	97 (54 - 174)	36.5 (20.7 - 156.7)	7.3 (6.5 - 8.3)	60.0 (51.2 - 68.8)
Subject 5	3.28 (0.65 - 16.51)	209 (75 - 585)	28.0 (11.9 - ∞)	6.2 (5.2 - 7.7)	53.9 (40.3 - 67.5)
Subject 6	3.85 (1.46 - 10.15)	242 (131 - 448)	24.7 (14.4 - 88.2)	6.1 (5.5 - 6.8)	53.4 (45.2 - 61.5)
Subject 7	5.48 (1.95 - 15.44)	4361 (798 - 23843)	19.6 (12.2 - 49.9)	4.0 (3.3 – 5.0)	62.2 (54.2 - 70.2)
Subject 8[b]	6.34 (0.56 - 72.27)	933 (3 - 331131)	18.0 (7.8 - ∞)	4.9 (2.6 - 34.4)	47.4 (31.3 - 63.5)

PRR_{48}: parasite reduction ratio after treatment standardized over a 48 hour period; CI: confidence interval. [a]Subject was not included in cohort-specific analysis because the break-point occurred later compared with other subjects in the cohort and there were insufficient data points to characterise the second phase slope. [b]Subject was not included in cohort-specific analysis because parasitaemia was very low throughout the study and the segmented regression had difficulties fitting to this subject.

M5717 resistance assessment methods and results (volunteer infection study)

Methods

Blood samples were collected from subjects who experienced recrudescence of parasitaemia following M5717 treatment (5 subjects). DNA was extracted using the QIAmp DNA blood mini Kit (Qiagen) as per the manufacturer instructions. The *PfeEF2* gene was PCR-amplified using outer primers flanking the entire 2.7 kb *PfeEF2* gene (Table S5). Two samples required a hemi-nested amplification of the initial PCR product whereby an inner primer pair consisting of the original 5' outer primer and a 3' primer situated 150 bp upstream of the original 3' primer was used for a second round of PCR amplification (Table S4). The PCR conditions for the initial amplification were: 95°C for 3 minutes, 45 rounds of 98°C for 20 seconds, 55°C for 30 seconds, and 68°C for 2.5 minutes, with a final extension of 3 minutes at 68°C. PCR conditions for the hemi-nest amplification were: 96°C for 3 minutes, 25 rounds of 96°C for 45 seconds, 54°C for 36 seconds, and 68°C for 2.5 minutes, with a final extension of 10 minutes. Agarose gel electrophoresis (1%) was used to confirm PCR product size. Sanger sequencing of PCR products was carried out by Genewiz using nine additional sequencing primers in addition to the PCR primers (Table S4). Sequences were aligned to WT *PfeEF2* and analysed using DNAStar's SeqMan Pro program. Electropherograms were visually inspected to identify mixed sequences indicating multiple subpopulations.

Table S5. Primers used for *PfeEF2* PCR amplification and sequencing

Primer Name	Sequence (5'→ 3')	PCR function
p7580	CTTTACGGTAGATCAAGTTCGTG	Outer Flank, Hemi-nest, Sequencing
p7582	AATATTACCGTGTATTTGTATGTAC	Outer Flank, Sequencing
p7584	CATTTACATATTTGTTGTAGTAGTGG	Hemi-nest
p7617	GTCATTATCTCTACATATACTGAC	Sequencing
p7618	CGTAATTGTGATCCTAATGGTCC	Sequencing
p7619	TCATTGTATCTGATCCAGTCGTC	Sequencing
p7620	ATGTTGTTAGTGGTGTGTATGGTG	Sequencing
p7621	CCAGAACCAAAAGATACAGTACC	Sequencing
p7622	AATCTACCCTTATCAGATGTAGG	Sequencing
p7623	TAAACGGTTGTGTTTGTTTGGTG	Sequencing
p7624	GAGTACCTAATTTCTGTTCTTCTG	Sequencing
p7870	TAGCTTCTGGTAAACCTTCAGC	Sequencing

Results

High quality two-strand sequencing coverage across the complete *PfeEF2* gene was achieved for all samples after amplification. Single nucleotide polymorphisms were observed in four of the five samples (Table S6). Of note, earlier work identified E134D and E134G mutations in M5717-pressured parasite lines that yielded 40 to 50 fold increase in the IC_{50} values relative to the starting drug-sensitive parental lines (Dd2 and 3D7, respectively). I183T was also observed in M5717-pressured 3D7 parasites, yielding a 5-fold IC_{50} increase. The S474R mutation was observed in a 7G8 mutant with a 35-fold IC_{50} increase. For residue 754, a P754A mutation was observed in M5717-pressured 3D7 parasites that showed a 40-fold IC_{50} increase. These mutants, generated in the Fidock lab, were reported in Baragana *et al.* 2015 (*Nature* 522: 315-20). That study also reported a minimum inoculum of resistance (MIR) of between 3×10^6 and 1×10^7 parasites. This MIR is similar to the value we obtain with the PfATP4 inhibitor cipargamin (Lee *et al*. 2016, *PloS One* 21;11:e0154166) and the dihydroorotate dehydrogenase (DHODH) inhibitor DSM265 (Phillips *et al*. 2015, *Sci Transl Med* 15;7:296ra111).

Table S6. Results of *PfeEF2* sequencing analysis

Subject ID	Nucleotide position	WT allele	Mutant allele	Codon change	Amino acid position	WT amino acid	Mutant amino acid	Note
**Cohort 1 (400 mg M5717)**								
Subject 3	400	G	C	GAA → CAA	134	E	Q	Mutant
Subject 8	2260	C	G	CCA → GCA	754	P	A	Mutant
**Cohort 2 (150 mg M5717)**								
Subject 1	1420	A	C	AGT → CGT	474	S	R	Mutant
Subject 2								Wild-type
Subject 6	549	C	G	ATC → ATG	183	I	M	Mutant

Measurement of M5717 in whole blood (single ascending dose and volunteer infection studies)

Human whole blood samples were diluted 1/1, v/v with 100 mM citric buffer and subsequently stored at -75°C ± 15°C. A method was validated for the quantification of M5717 in human whole blood and consisted of a protein precipitation extraction followed by high performance liquid chromatography with MS/MS detection. M5717-d8 was used as the internal standard.

Liquid chromatographic separation was performed at 40°C using a Waters Acquity UPLC HSS T3, 50 x 2.1 mm, 1.8 µm column. One of two gradient elution programs was performed using mobile phase A (acetonitrile) and mobile phase B (1000 mL water, 4 mL 2 M ammonium formate buffer, 1000 µL 98 % formic acid) as follows:

Gradient program 1 (flow-rate of 0.6 µL/min); 0 min (0% A, 100% B), 0.5 min (0% A, 100% B), 0.6 min (70%A, 30% B), 1.7 min (70% A, 30% B), 1.8 min (0% A, 100% B), 2 min (0% A, 100% B).

Gradient program 2 (flow-rate of 0.5 µL/min); 0 min (10% A, 90% B), 0.2 min (10% A, 90% B), 1.5 min (90%A, 10% B), 1.7 min (90% A, 10% B), 1.8 min (10% A, 90% B), 2 min (10% A, 90% B).

A Sciex Triple Quad 5500 or API 5500 mass spectrometer at unit resolution in the multiple reaction monitoring mode was used to monitor the transition of the protonated precursor ions, 463.2 m/z and 471.3 m/z, to the product ions, 234.1 m/z and 400.2 m/z, for M5717 and M5717-d8, respectively. Turbo ion spray ionisation was used for ion production.

Details of assay validation are presented in Table S7.

Table S7. Assay to determine M5717 concentrations in whole blood (diluted (1:1 with 100 mM citric acid)

Parameter			Result
Calibration range (lower limit is LLOQ)			1.00-2000 ng/mL
Selectivity (1 run)	Intra-assay	Accuracy (%bias)<br>Precision (%CV)	≤ 5.0%<br>≤ 5.6%
QC results (LLOQ)	Intra-assay	Accuracy (%bias)<br>Precision (%CV)	5.6%<br>11.5%
	Inter-assay	Accuracy (%bias)<br>Precision (%CV)	6.3%<br>7.2%
Extraction recovery (1 run)		For M5717	Low: 92.5%<br>Medium: 93.2%<br>High: 91.5%
		For M5717-d8	Medium: 90.5%
Carryover			<20% LLOQ on 2nd blank after ULOQ
Stability in whole blood (diluted 1/1 with citric acid)	Number freeze-thaw cycles<br>At ambient temperature<br>At −70°C		4: stable<br>Up to 1 hour<br>376 days: stable
Matrix effect			None
Stability in whole blood (ambient temperature)			1 hour: stable
Extract sample stability (ambient temperature)			24 hour: stable
Extract sample stability (5ºC)			4 days: stable

CV=coefficient of variation; ID=identification; LC-MS/MS=liquid chromatography coupled with tandem mass spectrometry; LLOQ=lower limit of quantification, ULOQ=upper limit of quantification.

General study information

Eligibility criteria for subject enrollment (single ascending dose and volunteer infection studies)

Inclusion criteria

1. Adult men and women of non-childbearing potential, 18 to 55 years of age (inclusive), with total body weight ≥ 50.0 kg and body mass index (BMI) between 19.0 kg/m^2 and 29.9 kg/m^2 (inclusive).

2. A female subject was eligible to participate if she was not breastfeeding and not a woman of childbearing potential confirmed at Screening by fulfilling one of the following criteria:

Postmenopausal defined as having amenorrhea for ≥ 12 consecutive months following cessation of all exogenous hormonal treatments, and increased follicle-stimulating hormone (FSH) > 40 mIU/mL.
Documentation of irreversible surgical sterilization by hysterectomy, bilateral oophorectomy, or bilateral salpingectomy. Tubal ligation alone was not sufficient.

3. A male subject had to agree to use a condom with or without spermicide during sexual activity with a female partner of childbearing potential and had made the female partner use a highly effective contraception (i.e., methods with a failure rate of less than 1% per year) during the treatment period and for at least 20 weeks after the last dose of IMP administration, and refrained from donating sperm during that period.

4. Healthy as assessed by the Investigator with no clinically significant abnormality identified on physical examination or laboratory evaluation and no active clinically significant disorder, condition, infection or disease that might pose a risk to subject safety or interfere with the trial evaluation, procedures, or completion.

5. Stable non-smokers for at least 6 months preceding Screening.

6. Able and willing to comply with restrictions on exposure to sunlight and to give written informed consent.

7. Additional for VIS part only: Did not live alone (from start of malaria inoculation until at least the end of the antimalarial drug treatment), were willing to provide contact details of a person living with them, and be contactable and available for the duration of the trial; no history of possible malaria exposure.

Exclusion criteria

1. History or presence of clinically relevant respiratory, gastrointestinal, renal, hepatic, hematological, lymphatic, neurological, cardiovascular, psychiatric, musculoskeletal, genitourinary, immunological, dermatological, connective tissue diseases or disorders.

2. History of any malignancy except surgically cured skin cancers.

3. History of relevant drug hypersensitivity, ascertained or presumptive allergy/hypersensitivity to the active drug substance and/or formulation ingredients; history of serious allergic reactions leading to hospitalization or any other allergic reaction in general, which the Investigator might consider to affect the safety of the subject and/or outcome of the trial.

4. History of alcoholism or drug abuse.

5. Liver functions tests:
above the laboratory reference range the day before IMP administration (Day -1) for SAD part.
≥ 3 × ULN on the day of IMP administration in VIS part.

6. Creatinine clearance < 90 mL/min as estimated using the Cockcroft and Gault equation at Screening.

7. Semi-supine blood pressure > 140/90 mmHg at Day -1 (this could have been repeated once).

8. Electrocardiogram showing a QT interval corrected for heart rate according to Fridericia's formula (QTcF) > 450 ms, PR > 210 ms, QRSD > 120 ms at Screening and predose.

9. Consumption of an average weekly intake of > 14 drinks/week for men or > 7 drinks/week for women. One drink was equivalent to 12 g alcohol = 5 ounces (150 mL) of wine or 12 ounces (360 mL) of beer or 1.5 ounces (45 mL) of 80-proof distilled spirits.

10. Positive for hepatitis B surface antigen, hepatitis B core antibody, hepatitis C antibody or human immunodeficiency virus I and II tests at Screening.

11. Positive for drugs of abuse, nicotine/cotinine or alcohol on Screening or (each) admission.

12. Use of any investigational drug in any clinical trial within 90 days from the last administration, or on extended follow-up in a clinical trial, even if the last administration of an IMP was > 90 days ago.

13. Donation or loss of more than 450 mL of blood within 90 days prior to Screening.

14. Excessive consumption of xanthine-containing food or beverages (> 5 cups of coffee per day or equivalent) or inability to stop consuming caffeine, from 48 hours prior to IMP administration until 48 hours after IMP administration.

15. Intake of grapefruit, Seville oranges, cranberries, star fruit or juices of these fruits, as well as quinine-containing food/beverages (e.g., tonic water, bitter lemon), within 14 days prior to IMP administration until the end of the residential period.

16. Ingestion of any poppy seeds within 24 hours prior to each Drug Abuse Screening.

17. Use of any prescribed medicine or over-the-counter drug (other than occasional ibuprofen [< 1 g] or paracetamol [< 1 g], or vitamins and minerals) within 2 weeks/5 times the half-life of the respective drug, whichever was longer, prior to the first administration of IMP.

18. Use of drugs and herbal remedies with enzyme inducing properties such as St. John's Wort, within 4 weeks before the first administration of IMP until the End of Trial visit.

19. Inability to refrain from taking up any new unaccustomed exercise from Screening until the End of Trial visit.

20. Inability to communicate reliably with trial site personnel or considered by the Investigator to be unable to or unlikely to cooperate with the requirements of the trial.

21. Having skin Type I, i.e., always burns and never tanned (pale peach complexion; blond or red hair; blue eyes; freckles; according to the Fitzpatrick scale.

Additional Exclusion Criteria for VIS part only:

22. Any history of malaria.

23. Participation in a previous malaria vaccine trial.

24. Participation in a previous human malaria challenge trial.

25. Had traveled to or lived (> 2 weeks) in a malaria-endemic area/region during the past 12 months. Malaria-endemicity was assessed by consulting https://map.ox.ac.uk/country-profiles/#!/.

26. Planned to travel to a malaria-endemic area/region during the course of the trial.

27. Had evidence of increased cardiovascular disease risk (defined as 5-year risk > 10%, for those > 35 years of age, as determined by the Australian Absolute Cardiovascular Disease Risk Calculator). Risk factors included sex, age, systolic blood pressure (SBP; mmHg), smoking status, total cholesterol and high-density lipoprotein cholesterol (mmol/L), and reported diabetes status.

28. Frequent headaches and/or migraine, recurrent nausea, and/or vomiting (> 2 times per month).

29. Presence of acute infectious disease or fever (i.e., sublingual temperature ≥ 38.0°C) within the 5 days prior to inoculation with malaria parasites.

30. History of splenectomy.

31. Subject unwilling to defer blood donations for 6 months.

32. Subject who had previously received a blood transfusion and/or tested positive for red blood cell antibodies.

33. Any corticosteroids, anti-inflammatory drugs, immunomodulators or anticoagulants in the past 3 months. Any subject who was receiving or had received immunosuppressive therapy, which included systemic corticosteroids including adrenocorticotrophic hormone or inhaled steroids in dosages which were associated with hypothalamic-pituitary-adrenal axis suppression or chronic use of inhaled high-potency corticosteroids.

34. Any recent (within 6 weeks from Screening) or current systemic therapy with drugs known to have potential antimalarial activity e.g., trimethoprim/sulfamethoxazole, tetracycline, doxycycline, erythromycin, clarithromycin, azithromycin, clindamycin, rifampicin, newer quinolones, benzodiazepines, flunarizine, fluoxetine, methotrexate, chloroquine, hydroxychloroquine.

35. Known allergy or adversity to one of the rescue medications proposed for the challenge trial which included known or confirmed deficiency for glucose-6-phosphate dehydrogenase (G6PD).

Blood sampling time points for M5717 concentration and parasitaemia measurements

Blood samples were collected at the following time points after dosing to determine the M5717 concentration in whole blood using liquid chromatography tandem mass spectrometry: 0.5, 1, 1.5, 2, 4, 6, 8, 10, 12, 16, 20, 24, 30, 36, 42, 48, 72, 84, 96, 120, 144, 192, 240, 384 (day 17), 504 (day 22), 768 (day 33), 1032 (day 44), and 1296 hours (day 55, cohort 1 of SAD only).

Blood samples were collected at the following time points after dosing in the VIS to determine the pharmacodynamics of parasite clearance by 18S qPCR: 4 (400 mg cohort only), 6 (150 and 800 mg cohorts only), 8 (400 mg cohort only), 12, 16, 20 (150 and 800 mg cohorts only), 24, 30, 36, 42 (150 and 800 mg cohorts only), 48, 54 (150 and 800 mg cohorts only), 60, 66 (150 and 800 mg cohorts only), 72, 84, 96, 108, and 120 hours. Blood sampling continued every one to three days to monitor for recrudescence and ensure clearance of parasitaemia following artemether/lumefantrine treatment.

Table S8. Key trial dates

SAD cohort	VIS cohort	Start Date	End date
Cohort 1 (50 mg)		12 Sep 2017	11 Nov 2017
Cohort 2 (100 mg)		21 Oct 2017	20 Dec 2017
Cohort 3 (200 mg)		16 Jan 2018	17 Mar 2018
Cohort 4 (400 mg)		20 Feb 2018	15 Apr 2018
	Cohort 1 (400 mg)	29 Mar 2018	15 Jun 2018
Cohort 5 (600 mg)		03 Apr 2018	22 May 2018
	Cohort 2 (150 mg)	11 Jun 2018	01 Aug 2018
Cohort 6 (1000 mg)		21 Aug 2018	08 Oct 2018
	Cohort 3 (800 mg)	8 Oct 2018	07 Dec 2018
Cohort 7 (1250 mg)		13 Nov 2018	04 Jan 2019
Cohort 8 (1800 mg)		19 Mar 2019	10 May 2019
Cohort 9 (2100 mg)		30 Apr 2019	14 Jun 2019
DATABASE LOCK		06 Aug 2019	
